# Neuroprotective effect and preparation methods of berberine

**DOI:** 10.3389/fphar.2024.1429050

**Published:** 2024-09-06

**Authors:** Yi-Xuan Sunhe, Yue-Hui Zhang, Rui-Jia Fu, Ding-Qiao Xu, Yu-Ping Tang

**Affiliations:** Key Laboratory of Shaanxi Administration of Traditional Chinese Medicine for TCM Compatibility, Shaanxi University of Chinese Medicine, Xianyang, Shaanxi, China

**Keywords:** berberine, dosage form, neuroprotective, mechanism, pharmacology

## Abstract

Berberine (BBR) is a natural alkaloid, which has played an important role in the field of medicine since its discovery in the late 19th century. However, the low availability of BBR *in vivo* prevents its full effect. In recent years, a large number of studies confirmed that BBR has a protective effect on the nervous system through various functions, yet the issue of the inability to systematically understand the protection of BBR on the nervous system remains a gap that needs to be addressed. Many existing literature introductions about berberine in neurodegenerative diseases, but the role of berberine in the nervous system goes far beyond these. Different from these literatures, this review is divided into three parts: preparation method, mechanism, and therapeutic effect. Various dosage forms of BBR and their preparation methods are added, in order to provide a reasonable choice of BBR, and help to solve the problem of low bioavailability in treatment. More importantly, we more comprehensively summarize the mechanism of BBR to protect the nervous system, in addition to the treatment of neurodegenerative diseases (anti-oxidative stress, anti-neuroinflammation, regulation of apoptosis), two extra mechanisms of berberine for the protection of the nervous system were also introduced: bidirectional regulation of autophagy and promote angiogenesis. Also, we have clarified the precise mechanism by which BBR has a therapeutic effect not only on neurodegenerative illnesses but also on multiple sclerosis, gliomas, epilepsy, and other neurological conditions. To sum up, we hope that these can evoke more efforts to comprehensively utilize of BBR nervous system, and to promote the application of BBR in nervous system protection.

## 1 Introduction

Berberine (BBR) was first discovered from the bark of *Xanthoxylon clava*. In the past, BBR was isolated from plants such as Berberidaceae, Ranunculaceae, Rutaceae, Menispermaceae, Papaveraceae, Loganiaceae, and Rhamnaceae. Nowadays BBR can be synthesized artificially (Zhang and Shen, 2023). Numerous studies on the pharmacological effects and associated mechanisms of BBR have been carried out recently in many countries. These studies have revealed that BBR has unique impacts and pharmacological activities on the cardiovascular system, nervous system, and endocrine system. These effects can lower blood lipids and protect the cardiovascular system, produce anti-anxiety effects by influencing brain neurotransmitters, and improve insulin sensitivity in the treatment of diabetes. The application potential of BBR in both the prevention and treatment of cardiovascular, cerebrovascular, nervous system, and other important disorders cannot be ignored (Hu and Mo, 2017).

As the worldwide population ages, hundreds of thousands of elderly people suffer from neurological issues. The majority of medications have adverse effects for central nervous system disorders (Dording and Boyden, 2019). But as a natural alkaloid, BBR has fewer adverse effects and offers some benefits in the medical management of central nervous system disorders. Researchers reported that BBR can penetrate the blood-brain barrier, reduce the permeability of the blood-brain barrier, and protect the integrity of the blood-brain barrier, thus maintaining the homeostasis of the central nervous system (Ma et al., 2010; Song et al., 2022) and is more suitable for brain diseases. Through its unique regulation mechanism, BBR have a great effect on regulating the nervous system (Ding et al., 2021). Moreover, BBR has a ameliorative impact on neurodegenerative diseases (Alzheimer’s disease, Parkinson’s disease, and Huntington’s disease) and psychiatric diseases (depression, anxiety, schizophrenia). To better investigate and develop the use of BBR in central nervous system illness, we elucidated the extraction method of BBR and dosage forms of BBR, as well as its pharmacological effects on inflammation, oxidative stress, apoptosis, autophagy and angiogenesis were described. In addition, it summarizes how BBR affects various signaling pathways to regulate the processes of central nervous system diseases.

### 1.1 Physicochemical properties and pharmacokinetics of BBR

**FIGURE 1:**
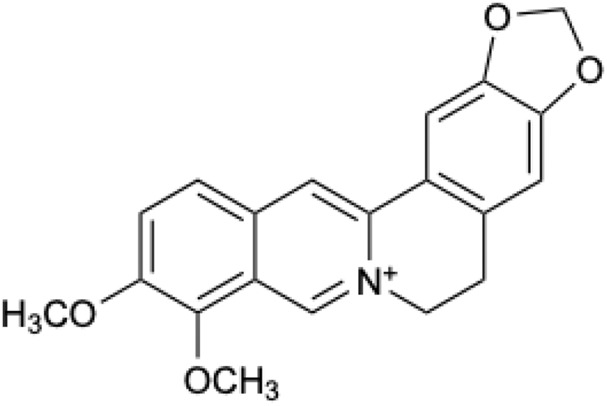
Chemical structure of BBR.

BBR is a quaternary amine alkaloid with unique physical and chemical properties (Li et al., 2020). The molecular formula of BBR is C_20_H_18_NO_4_. BBR is a faint yellow acicular crystal, the melting point is 204.8–205.4°C. (The chemical structural formula of BBR is seen in **Figure 1**). BBR has high solubility in hot water, slight solubility in cold water and cold ethanol, and almost insoluble in chloroform and ether. Under physiological conditions, BBR mainly exists in ionized form and is easy to self-accumulate in the acidic environment of the gastrointestinal tract. The solubility at pH 1.2 (HCl) is 1/20 of that at pH 7.0 (Spinozzi et al., 2014). Due to the lipophobic nature of BBR, its passage through intestinal cells is obstructed. Its effective permeability coefficient in rat intestinal mucosa was 0.178 × 10^−4^ cm s^-1^, which confirmed its low permeability (Chen et al., 2011; Zhou, J. X. et al., 2022).

BBR is poorly soluble. BBR that is absorbed in by the gut can also be expelled back into the intestinal lumen by the action of P-glycoprotein (P-gP). BBR is the substrate of the efflux transporter P-gP, which makes BBR difficult to be absorbed orally and shows a very low bioavailability (<1%) in both animals and humans. Chen et al. studied that the absolute bioavailability of oral pathway in rats was 0.68% (Chen et al., 2011). In addition, P-gP inhibitors could increase the absorption of BBR by 6 times in rats, indicating that P-gP contributed to intestinal malabsorption of BBR (Pan et al., 2002). Liu et al. discovered that after the rats were given BBR intragastric administration, approximately 1/2 of the BBR was eliminated through the gastrointestinal tract, while the remaining 1/2 was excreted through the intestinum tenue, resulting in the oral absolute bioavailability of rats is 0.36% (Liu et al., 2010). It was found through experimental determination that first pass elimination of the intestine of BBR was the main obstacle to its oral absolute bioavailability. The liver’s significant extraction and distribution of BBR might have led to its low concentration in rat plasma. After oral administration, BBR can be widely distributed in liver, kidney, brain, heart and other parts (Tan et al., 2013), and can penetrate the blood-brain barrier and rapidly distribute to the thalamus (Wang et al., 2005). A large amount of scientific evidence shows that BBR is metabolized by demethylation, glucuronidation and/or sulfonation (Ahmed et al., 2015), and the pharmacological activity of the metabolite is consistent with BBR (Wang et al., 2017). In both human and rats, the active metabolites of BBR are mainly divided into the following four kinds: berberrubine (M1), thalifendine (M2), demethyleneberberine (M3), jatrorrhizine (M4) (Liu et al., 2009). One study discovered that M1, M3, and M4 can directly cross the blood-brain barrier to perform a neuroprotective effect (Pan et al., 2024), but whether M2 can cross the blood-brain barrier remains unknown. In addition, the metabolites of BBR are all P-gP substrates, and the order of binding strength is as follows: M1 < BBR < M4 < M2 < M3. The higher the binding affinity, the more difficult the transport of the compound is (Zhang et al., 2019). Therefore, the oral bioavailability of M1 may be better than BBR. Not only that, BBR metabolites also showed higher concentrations in plasma, and the lipophilicity of M1 was higher than BBR (Wang et al., 2015), this suggests that the study of the metabolically active components of berberine may provide an opportunity to further improve the efficacy of BBR. Finally, in terms of excretion, BBR is mainly excreted through urine and bile in the form of metabolites. In addition, BBR has high safety, rarely significant adverse reactions in clinical trials, and its metabolite structure can remain relatively stable after entering the brain, suggesting that it can play a therapeutic role in nervous system diseases (Wang, X. J. et al., 2024).

### 1.2 Extraction method of BBR

BBR extraction methods mainly include Acid water extraction, Alkali water extraction and Alcohol extraction, of which acid water extraction and alkali water extraction are simple, low cost, but still have problems such as: low extraction rate, easy to corrode equipment, unsafe, not eco-friendly. Alcohol extraction methods include Microwave-assisted extraction, (Soxhlet) reflux extraction, Flash extraction, Ultrasonic extraction, etc., which not only has low solvent restriction and can be used repeatedly, but also can save energy and environmental protection, high safety, simple operation, and high extraction efficiency. However, ultrasonic extraction methods require large investment in equipment (Yang, Q. Z. et al., 2015), and the specific content of BBR extraction method is shown in Table 1.

**TABLE 1:** Different extraction methods of BBR.

Method		Procedure	Characteristic	Reference
Acid water extraction		The raw materials are soaked in a multiple amount of sulfuric acid water for 24h, the pH of the filtrate is adjusted to 10–12 with lime milk, filtration, the pH of the filtrate is 2–3 controlled by hydrochloric acid solution, and the refined salt is added, completely dissolved, placed overnight, suction filtration to obtain crude product	The extraction method is simple and low cost, but the extraction rate is low, unsafe, not environmentally friendly, easy to corrode equipment	Liao, Z. X. et al. (1998), Pang, X. X. and Xu, W. L. (1996)
Alkali water extraction		Add the raw materials into the lime milk, stir evenly, soak in saturated lime water for 6h, percolation, control the flow rate, add solid salt into the percolate, filtration, precipitate, dissolve in hot water, filter while its hot, add hydrochloric acid to adjust the pH to 2, stewing, filtration, wash the precipitate with water until neutral, suction filtration to obtain crude product	Compared with acid water extraction and alcohol extraction, the extraction efficiency is relatively better. However, due to the extensive use of lime milk, it may cause partial component loss	Cheng, Y. M. and Chen, R. H. (2007), Yin, R. L. et al. (2000)
Alcohol extraction	Microwave-assisted extraction	Soak the raw material powder with ethanol, recover most of it, concentrate the rest, filtration, salt-out acid extraction, precipitation, stewing, obtain crude product	Fast heating, uniform heating, easy to operate. But the research of extraction technology is still in the initial stage, and the parameters in the extraction process, such as the physical properties of medicinal materials, comminution degree, the content of free water or bound water, all have an impact on the extraction rate	Yang, Q. Z. et al. (2015), Deng, Y. H. et al. (2002)
	Flash extraction	Same as above	The extraction speed is fast, suitable for most solvents, high efficiency and energy saving, and easy to operate. Due to the small amount of herbs added at one time, it will be difficult to filter due to excessive water-soluble components, increased viscosity, suspension and emulsification	Liu, Y. Z. (2007), Qin, Z. F. and Li, H. Y. (2005)
	Ultrasonic extraction	Take raw material powder, add sulfuric acid solution, soak for 24h, ultrasonic treatment for a period of time, filtration. Take the filtrate, dilute it with water and shake well	Ultrasonic extraction technology can use the strong vibration generated by ultrasonic wave to accelerate the active ingredients into the solvent, so as to improve the extraction rate and avoid the influence of high temperature on the extracted ingredients. But it increases the difficulty of equipment investment and operation	Guo, X.W. et al. (1995), Wu, B. H. (2004), Ong et al. (2000)
Enzymatic extraction		The raw materials were pretreated by adding the enzyme solution, adding lime water, ultrasound, filtration, adding hydrochloric acid to adjust the pH to 2–3, adding salt and placing overnight, suction filtration to obtain the product	The extraction temperature of enzyme reaction is low, which can significantly increase the yield	Liang, B. L. and Zhou, M. J. (2006)
Microextraction		-	Less consumption, less funds, less environmental pollution, safety, short time But compared with the conventional chemical experiment, the yield is lower	Cheng, Y. M. and Chen, R. H. (2007)
Semi-bionic extraction		Take the raw materials and add water in a certain proportion and decoct for 3 times, take pH = 5.5 as the first decoction, take pH = 10 as the second and third decoction, repeat twice, combine the decoction, filtration, concentrate, add talc powder, stand, centrifuge, constant volume, and get the product	More active ingredients can be extracted and retained, shortening the production cycle and reducing costs	Lin, H. B. et al. (2004), Sun, X. M. et al. (1996), Zhang, Z. W. and Sun, X. M. (1995)
Supercritical CO_2_ extraction		The supercritical fluid is contacted with BBR to dissolve it fully, and then the supercritical fluid CO_2_ is changed into a gas by reducing pressure and heating up, and the BBR is precipitated	Compared with the traditional solvent extraction method, it has the advantages of low temperature, high speed, high efficiency, good pharmacological effect and lower toxicity	Liu et al. (2006)
Aqueous two phase extraction		The crude extract of raw materials was taken and added into (NH_4_)_2_SO_4_/PEG400 two-phase aqueous solution, centrifuged into two phases, and read the volume of the two phases, and the BBR concentration in the two phases was calculated to obtain its extraction rate	The extraction conditions are mild, the extraction phase does not contain polymer with large viscosity, and the phase separation is clearer and faster. The traditional two-phase aqueous system is generally polyethylene glycol - glucan, polyethylene glycol - inorganic salt, etc., most of them have large viscosity, hard to volatilize, and subsequent separation is more complicated	Yang, Q. Z. et al. (2015), Xie, T. et al. (2008), Wen et al. (2011), Li, M. Q. et al. (2006)
High pressure hot water extraction		The raw material is reflow with ethanol, filtration, concentrated and fixed volume, treated with high pressure hot water, and precipitated by recrystallization after standing and filtering	More simple than pressurized fluid extraction, the extraction rate is high, in the appropriate pressure range, as the pressure rises, the extracted component content increases	Ong and Len (2003)
Liquid-membrane extraction		Pour the liquid film into the mother liquor, stir at a slow speed for 10 min, separate the liquid film layer that has absorbed BBR with the separator funnel, pour into the beaker, repeat twice. After that, the film is broken in the constant temperature water bath, standing, filtering, constant volume and sampling	Improve the separation and concentration effect, without a lot of pretreatment, easy to achieve industrialization, low energy consumption, less chemical consumption, no secondary pollution, better economic benefits	Wang, D. J. et al. (2006)

### 1.3 The dosage form of BBR

BBR has a broad application prospect and is mainly used in the form of hydrochloride in clinic. However, due to the low solubility of hydrochloride, its popularization and use are limited. At the same time, the bitter taste of BBR and other reasons also make it difficult to swallow, affecting the compliance of patients. Therefore, the design of different dosage forms has been endless, including liposomes, β-cyclodextrin inclusion complex, dropping pills, microspheres, microemulsions, solid lipid nanoparticles, targeting drug delivery system, etc. (Wu, J. D. et al., 2013), the specific contents are shown in Table 2.

**TABLE 2:** Different dosage forms of BBR.

Dosage form	Definition	Procedure	Characteristic	Reference
Liposome	The superminiature spherical carrier preparation is prepared by encapsulating the drug in the middle of the thin film formed by the lipid double molecular layer	The ratio of phospholipid to cholesterol was 3∶1, the ratio of drug to lipid was 1∶15, the mass concentration of phospholipid was 30 g/L, and the pH of external aqueous phase was 7.0	It has remarkable sustained release properties *in vitro*	Wang et al. (2013)
		The liposomes were stable and reliable when the ratio of BBR hydrochloride to polysorbate 80 was 1∶0.4 and the ratio of soybean lipid (soybean phosphate) was 1∶20	Targeted, long-acting, low-toxicity, slow-release, non-immunogenic and protective encapsulation drugs, can increase gastrointestinal absorption	Xu et al. (2004)
		The liposomes prepared by injection method had uniform size, average particle size of 0.79μm, high encapsulation rate, high purity, and simple and easy content detection	Easy to operate,the synthesized BBR hydrochloride has a high purity and provides a reliable theoretical basis for the industrial production of water-soluble drug sustained-release injection	Jin et al. (2011)
		BBR hydrochloride liposomes were prepared by active drug loading method, and the liposomes were separated by cation exchange resin method. The liposomes obtained by this method had small particle size and high encapsulation rate, and the encapsulation rate was different with different dosing sequence. The optimal pH value of external water phase is 6.8	The mixing sequence of blank liposome, NaHCO_3_ solution and BBR hydrochloride solution has a certain effect on the encapsulation rate	Deng et al. (2004)
β- cyclodextrin inclusion complex	Oligomer consisting of 7 glucose units bound by 1, 4-glucoside bonds	The dosage of BBR was 0.030g, β-cyclodextrin was 2.0g, the inclusion time was 90min	The antibacterial ability of liposome is stronger than BBR alone, which can reduce drug dose and stimulation, prolong drug action time and improve drug efficiency, which has great economic and practical value in pharmaceutical engineering	Li et al. (2003)
		It was prepared by saturated aqueous solution method and orthogonal test method. The optimum process was as follows: temperature was 80°C, inclusion 2h, and the ratio of host and guest molecules was 4∶1 (g/g)	It can improve the solubility of insoluble drugs, improve bioavailability and cover up odors	Qi, (2010)
Dropping pill	drug is heated and mixed with the matrix, insoluble condensing agent is dropped, and the molten drop shrinks into a pill in the condensate and then condenses into a solid state	PEG1000+PEG4000 (1∶1) was used as the matrix, the drug-matrix was 1∶4, the material temperature was 95°C, the dimethylsilicone oil was used as the coolant, the coolant temperature was 5°C, the drip diameter was 3mm, the drip rate was 50 drops/min, the drop distance was 6 cm	This process provides a reference method for reforming some insoluble drug dosage forms	Chen and Zhou, L. J. (2008)
		The ratio of drug and matrix (PEG6000) is controlled at 1∶4, the drop rate in the drip process is controlled at 40 or 50 drops/min, the temperature of the liquid is 70 or 85–90°C, the temperature in the middle of the condensing tube is 6–8°C, the temperature in the bottom of the condensing tube is −2°C, the drop distance is 5 or 7cm, and the diameter of the dropper is 1.2–1.5 mm or 2 mm. The height of condensing column is 90–100 cm	The prepared dropping pills have small dissolution time, good appearance quality and small difference in pill weight, which meet the quality requirements of dropping pills. The method has certain value for the industrial production and clinical application of dropping pills	Luo, Y. N. and Qin, S. M. (2011)
Microcapsules	The solid, liquid or gaseous substance is coated in a small, closed system by means of physical chemistry, chemistry and other methods	Polyacrylic acid resin Ⅳ was dissolved in an appropriate amount of acetone, the raw material was added (1:1), and the suspension drops were added to an appropriate amount of liquid paraffin, stirred to heat up, filtered, and the formed microcapsules were obtained by washing and drying	Improving drug bitterness without altering pharmacokinetic properties, simple preparation process, large drug load, high encapsulation rate, stability, and good industrial application prospect	Liu, J. et al. (2004)
		BBR-hydroxypropyl methylcellulose phthalate (HPMCP) (1:7), HPMCP-acetone ethanol mixture (1:20), acetone ethanol mixture - liquid paraffin wax (1:5), sorbitan oleate - liquid paraffin (6:100)	It can reduce the frequency of administration, increase the adaptability of animals to drugs, and avoid drug inactivation in the stomach and reduce stomach irritation	Wang et al. (2014a)
		The mass ratio of core to material was 1∶3, the mass fraction of acacia and gelatin were both 2.50%, the stirring speed was 200 r/min, and the temperature was 53°C	Improve the taste and reduce the stimulation to gastric mucosa. But the drug carrying capacity of microcapsules is lower than fluidized bed coating	Yu, Y. et al. (2015)
Microspheres	A spherical or sphere-like entity in which a drug is dissolved and dispersed in a matrix skeleton made of polymer materials	The volume ratio of anhydrous ethanol to liquid paraffin was 1:8, the mass fraction of sorbitan oleate was 2%, the dosage of BBR was 800mg, the dosage of ethyl cellulose and carbomer were 500mg, and the mass ratio of ethyl cellulose and carbomer was 1:1	slow release	Jiang et al. (2015a)
		The dosage of BBR and polylactic acid was 0.02∶0.3. Water and oil phase volume ratio of 10∶100. The concentration of emulsifier PVA is 3%	Improve the encapsulation rate of BBR, Improve drug efficacy	Liu, Y. J. and Zhang, L. Y. (2008)
Pellets	Spherical, spheroid particle size is less than 2.5mm, composed of powder and excipients multi-drug release system	The solution of sodium alginate of BBR hydrochloride, sodium bicarbonate and chitosan is added to the solution of calcium chloride containing acetic acid	Can float in the stomach; Slow release	Su, Q. et al. (2023)
		1% carboxymethylcellulose sodium (CMC-Na) is the wetting agent, the extrusion rate is 40r/min, the rotating speed is 800r/min, and the rotating time is 2.5min	The process is simple, the production efficiency is high, the repeatability between batches is good, and the batch preparation can be scaled up, and it is suitable for the drug with less water solubility	Yu, L. M. (2012)
Nanoemulsion	Particle size of 10–100 nm,the emulsion droplets are dispersed in another liquid to form a colloidal dispersion system	BBR hydrochloride nanoemulsion was prepared by pseudo ternary phase diagram with isopropyl myristate as the oil phase, polyoxyethylene castor oil as the surfactant and glycerin as the cosurfactant	Compared with traditional tablets, capsules and aqueous solutions, it has higher antibacterial activity against *Escherichia coli*, *Salmonella*, *Staphylococcus aureus* and *Streptococcus* agalactis, reduces the dose and stimulating effect of BBR hydrochloride, prolongs the action time of the drug and improves the therapeutic effect of the drug	Sun and OY (2007)
Nanoparticles	A novel drug carrier with a particle size of 1–1000 nm	Gelatin was 10 g/L, the volume fraction of coagulant was 81.25%, the titration rate was 2 mL/min, the stirring rate was 600r/min, the mass ratio of BBR hydrochloride to gelatin was 2∶4, and the volume fraction of crosslinking agent was 10%	slow release	Li, X. D. et al. (2015)
		A sodium tripolyphosphate solution with a concentration of 1.5 mg/mL was slowly dropped into a chitosan solution with a concentration of 0.5 mg/mL BBR, and stirred for 10 min to obtain BBR chitosan nanoparticles	Improve drug bioavailability	Lin, A. H. et al. (2007)
Tablet	Orally disintegrating tablets	BBR hydrochloride was coated with acrylic resin, and the mass ratio of drug to coating material was 1:0.8. The drug microcapsules formed by coating were then pressed into tablets with 6% crospovidone and 15% microcrystalline cellulose tablets	Conceal the bitterness of BBR, easy to use, fast disintegration speed	Hu et al. (2013)
	Gastric floating tablets	Weigh BBR and various auxiliary materials according to the prescription, grind and pass 80 mesh sieve respectively, fully mix with equal amount method, add magnesium stearate, mix well. Press the tablet, the hardness of the tablet is controlled at 5 kg	It has good floating characteristics and drug release performance, and the production process is simple, and may be an alternative dosage form for the treatment of gastrointestinal diseases	Ji et al. (2017)
	Colon-location tablets	The multi-layer film coating technology of rolling coating machine was adopted. Hydroxypropyl methylcellulose was used as isolation layer, pH-sensitive acrylic resin mixed coating solution was used as enteric-soluble layer, permeable acrylic resin mixed solution was used as slow-release layer, triethyl citrate was used as plasticizer and talc powder was used as anti-stick agent. Isolation layer weight increased by 1.2%; Enteric layer composition (1:5), coating weight increased by 4%; Slow-release layer composition (1:1), coating weight increase 2%	It can improve the operability and is suitable for industrial production, and makes a beneficial exploration for the further study of colon targeted drug delivery system of traditional Chinese medicine	Liu, X. et al. (2008)
		An appropriate amount of microcrystalline cellulose was added to BBR hydrochloride, pectin and guar gum enzyme-controlled skeleton materials were pressed into a skeleton core and then coated with intestinal coating to make a colon-positioned skeleton coated tablet. The ratio of pectin to guar gum was 1:1, and the weight of enteric coating was increased by 3.8%	It can make the drug release less than 20% in artificial intestinal fluid in 5h, and close to 80% in simulated colon environment in 6 h	Xiao, Y. et al. (2008)
Gels	Ophthalmic	Appropriate amount of BBR hydrochloride and sodium chloride were dissolved in distilled water, Poloxamer was added (25% Poloxamer 407% and 4.19% Poloxamer 188), completely dissolved, and 0.02% benzalkonium bromide was added to make BBR hydrochloride ophthalmic gel	The problems of short retention time and low bioavailability of conventional ophthalmic liquid preparations were solved. The problems of poor spreading and difficult dose control of common ophthalmic gel were overcome	Hao, J. F. et al. (2010)
	Nasal	Weigh BBR hydrochloride, add Tween-80 in the mortar, homogenize, add glycerin and 0.2% Carbomer-980 aqueous solution, add 10% (V/V) triethanolamine solution, adjust pH6.0 ∼ 7.0, quantify, stir evenly, vacuum debubbling for 12 h	simple prescription, stable quality and release	Wang et al. (2014b)
Powders		The optimum process of BBR hydrochloride/montmorillonite composite powder is as follows: feed ratio 1:3, temperature 70°C, reaction time 2 h	It has good inhibitory effect on *Escherichia coli*	Sun et al. (2017a)

The aforementioned list includes all possible dose forms, preparation methods, and administration characteristics of berberine; nevertheless, the ones that may be useful in neurological illnesses are of greater significance. Most neurological diseases, including multiple sclerosis, Parkinson’s disease, Alzheimer’s disease, epilepsy, and cerebral infarction, require long-term medication. The benefits of slow-release dosage forms include decreased frequency of administration, stable drug concentration, and fewer side effects related to peak blood drug concentration (Andrade, 2015). Therefore, berberine is more suitable for long-term drug use when the dosage form is liposome, microsphere, nanoemulsion, and nanoparticles. In addition, the poor water solubility and low bioavailability of berberine is also a problem that must be paid attention to. Wang et al. ’s study showed that when Berberine is prepared as an inclusion compound of β-cyclodextrin, specific intermolecular interactions can be formed between Berberine Hydrochloride and β-cyclodextrin, and this dosage form greatly improves the solubility of berberine, thereby improving its bioavailability (Wang et al., 2020). Furthermore, the drug absorption velocity and absorption rate are enhanced when the dose forms are microspheres and nanoparticles because of their small particle sizes, which enhances bioavailability. The creation of berberine dosage forms is crucial for the nervous system’s application, and suitable dosage forms enable berberine to fulfill its therapeutic function and enhance its healing effect.

## 2 Pharmacological mechanism of neuroprotective effect of BBR

### 2.1 Anti-oxidative stress

**FIGURE 2:**
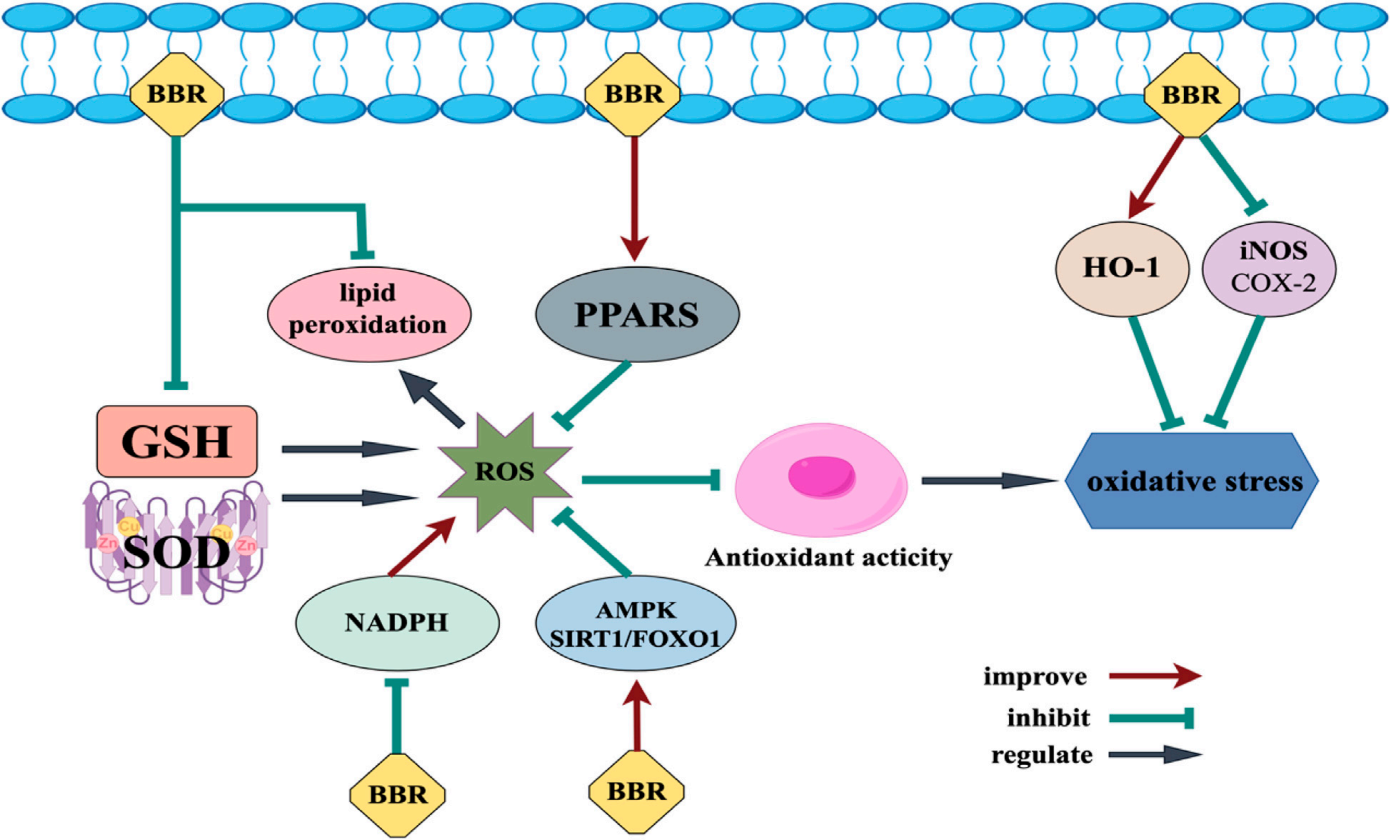
Mechanism of BBR against oxidative stress.

Oxidative stress occurs because of an excessive accumulation of reactive oxygen species (ROS), leading to an imbalance between oxidants and antioxidants, which in turn leads to the production of many neurological diseases (Li et al., 2020). During oxidative stress, an overabundance of reactive oxygen species (ROS) and/or reactive nitrogen species (RNS) can lead to lipid peroxidation, protein oxidation, protein nitration, and sugar co-oxidation. This can cause damage to the plasma membrane of nerve tissue, destruction of the cytoskeleton, and mutation of nucleic acids (Barnham et al., 2004; Uttara et al., 2009). The generation of ROS is related to glutathione (GSH), superoxide dismutase (SOD) and other chemicals (Li, H. Y. et al., 2024). Once ROS is excessive and inhibits the antioxidant activity of cells, oxidative stress will occur (Cheng et al., 2022), the mechanism of BBR against oxidative stress is shown in Figure 2. BBR has the potential to reduce ROS production and protect nerve cells from oxidative damage. Nicotinamide adenine dinucleotide phosphate (NADPH) oxidase is a primary producer of ROS, and BBR can alleviate oxidative stress by decreasing the production of NADPH oxidase (Ma et al., 2018). BBR effectively inhibits lipid peroxidation while enhancing glutathione content and superoxide dismutase activity in cells (Sadeghnia et al., 2017). BBR can effectively decrease the generation of ROS in both cytoplasmic and mitochondrial cells (Sun Y. et al., 2017). This reduction is likely due to the activation of Adenosine monophosphate (AMP)-activated protein kinase (AMPK) and sirtuin1 (SIRT1)/forkhead box O1 (FOXO1) pathways (Li, H. R., 2024). The antioxidative stress activity of BBR is also associated with the transcription factor PPAR, which is a transcription factor induced by ligands. Activation of PPARs can play an antioxidant role, And BBR can activate PPARδ to clear ROS and exert neuroprotective effects (Shou et al., 2022). BBR may also exert antioxidant effects by lowering inducible nitric oxide synthase (iNOS), cyclooxygenase-2 (COX-2), and boosting HO-1.

### 2.2 Anti-neuroinflammation

Neuroinflammation is the inflammation of nerve tissue, resulting from various sources such as traumatic brain damage and autoimmune. Although neuroinflammation can start in different trigger sites, it all has one thing in common: microglia and astrocytes are constantly activated (Perry and Holmes, 2014), Figure 3 shows how BBR works to reduce neuroinflammation. BBR can prevent neuroinflammation and possibly be used as a candidate drug to treat illnesses of the central nervous system that are caused by inflammation. BBR may provide neuroprotective effects by decreasing the generation of several neurotoxic compounds by activated microglia (Nam et al., 2010), BBR has been observed to effectively suppress the activation of NF-κB and the phosphorylation of Akt, p38, and extracellular regulated protein kinases (ERK). It is suggested that BBR may hinder the inflammatory response of microglia by reducing the PI3K/Akt and mitogen-activated protein kinase (MAPK) pathway(Wan, J. S. and Zhang, M. R., 2018). BBR inhibits the pro-inflammatory reaction by stimulating AMPK in BV-2 microglia. Additionally, BBR notably reduces the expression of iNOS and COX-2 in BV-2 microglia induced by LPS or interferon (IFN)-γ. It also hinders the synthesis of nitric oxide. Furthermore, BBR has the ability to normalise inflammatory factors. It can effectively decrease the generation of pro-inflammatory cytokines TNF-α and IL-1β, as well as the manifestation of IL-6 in BV2 cells that have been activated by LPS (Lu et al., 2010; Zhang et al., 2016a). BBR also decreases the production of inflammatory factors and GFAP, while suppressing Sphk1/S1P signalling and stimulating CREB signalling (Cheng et al., 2022).

**FIGURE 3:**
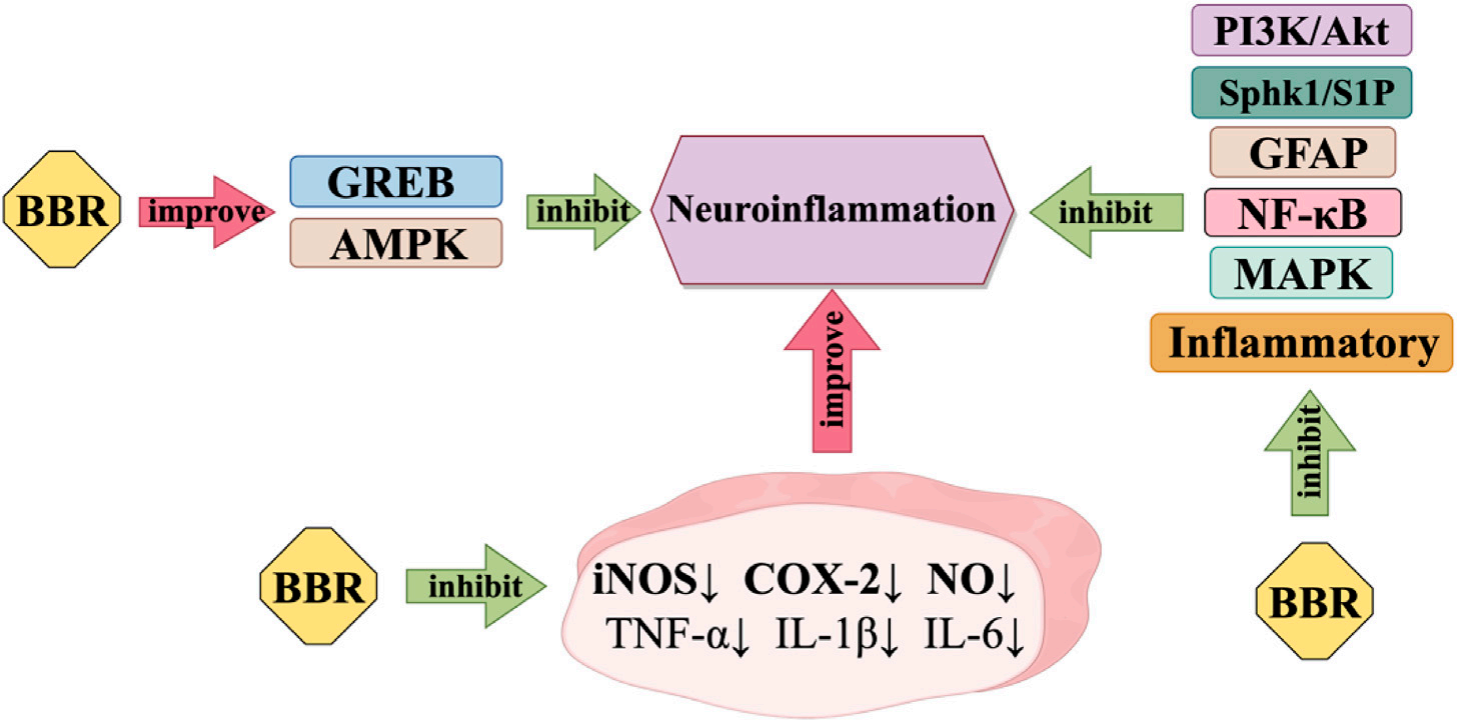
Anti-neuroinflammatory mechanism of BBR.

### 2.3 Regulation of apoptosis

**FIGURE 4:**
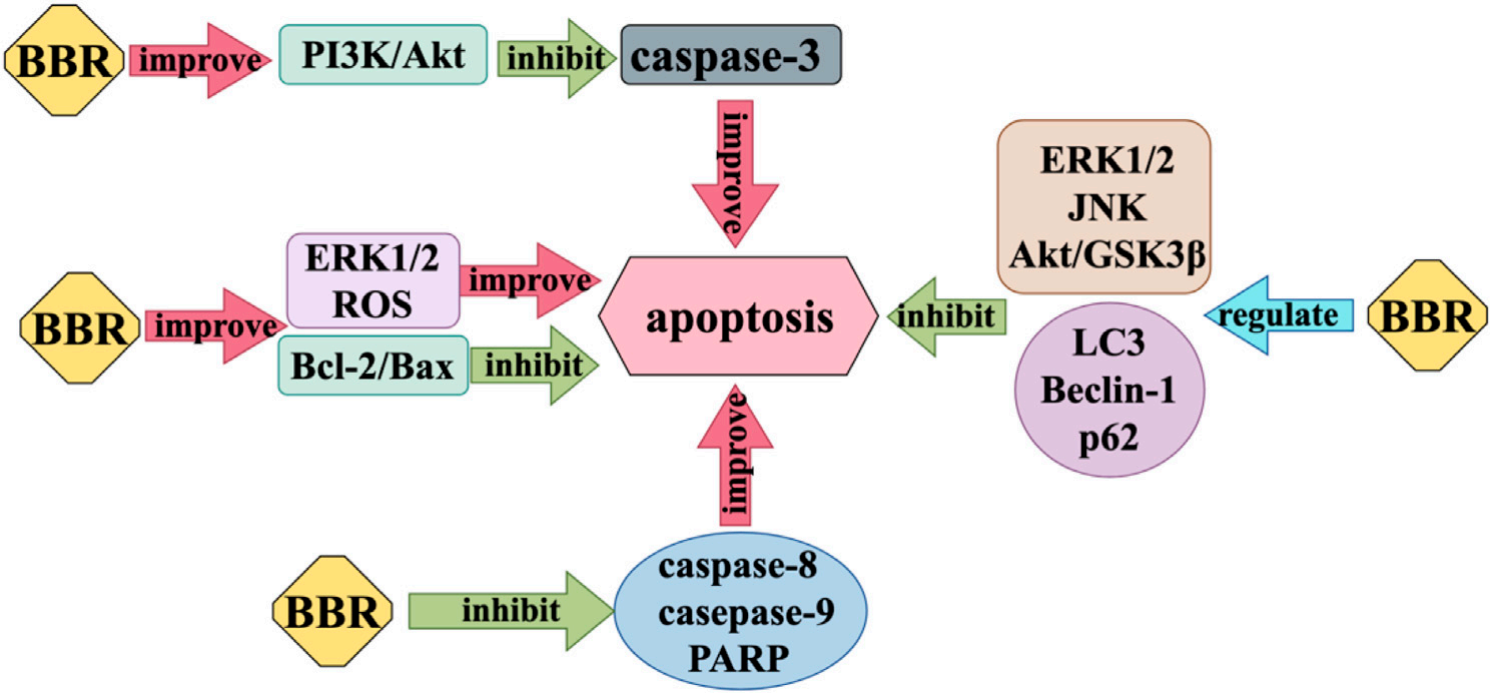
Regulation of apoptosis mechanism of BBR.

Nerve cell injury can cause apoptosis to some extent, resulting in the loss of nervous system function. Apoptosis is one of the important autostable mechanisms in multicellular organisms (Li, M. and Lin, J., 2014). BBR exerts anti-apoptosis effects on nerve cells by inhibiting caspase-3, which acts as an executor of apoptosis, and increasing the expression ratio of anti-apoptotic protein B-cell lymphoma 2 (Bcl-2) and pro-apoptotic protein Bcl-2 associated X (Bax). BBR modulates the activity of many proteins involved in autophagy, such as Microtubule-associated protein 1A/1B-light chain 3 (LC3), Beclin-1, and p62. It also affects the function of apoptosis regulating proteins, including caspase 3, caspase 8, caspase 9, poly ADP-ribose polymerase (PARP), and Bcl-2/Bax, which play a role in inhibiting neuronal apoptosis (Zhang et al., 2016b). Liang et al. found that BBR can inhibit the cytotoxicity of Aβ25-35 on neuron cells, reduce the activity of various caspase proteins, increase Bcl-2/Bax, and have anti-apoptotic effects (Liang et al., 2017). Most studies (Kim et al., 2014a; Kim et al., 2014b; Simões Pires et al., 2014; Hsu et al., 2012) believe that BBR can regulate the activity of subunit p55γ promoter and activate PI3K/Akt pathway by enhancing PI3K kinase, and then inhibit the activity of Bad, a positive regulator of apoptosis, and reduce the production of pro-apoptotic caspase-3 (Hu et al., 2012). But Simoes et al. propose that the Akt/GSK3β pathway, ERK1/2 pathway, and JNK pathway play an important part in the neuroprotective action of BBR against apoptosis (Simões Pires et al., 2014). However, in addition to the anti-apoptosis effect of BBR, when it comes to the specific therapeutic effect of BBR on some nervous system diseases, it also has the pro-apoptotic effect of inducing disease cell apoptosis. For example, in the treatment of glioblastoma, BBR inhibits ER stress-induced apoptosis in T98G cells by generating ROS and mitochondria-dependent mechanisms (Eom et al., 2010), BBR can also induce apoptosis of U251 and U87 cells by significantly inhibiting the activation of ERK1/2 pathway (Rauf et al., 2021) (Figure 4).

### 2.4 Regulation of autophagy

**FIGURE 5:**
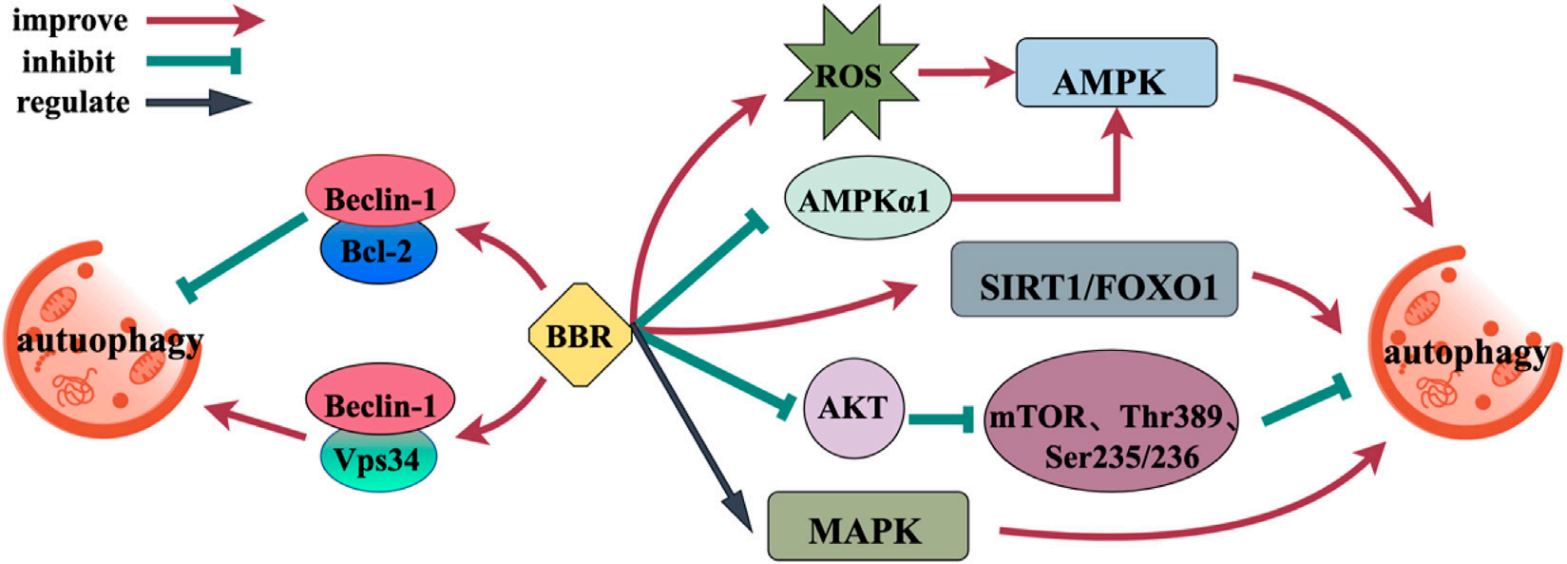
Mechanism of BBR regulating autophagy.

Upregulation of autophagy is a promising treatment approach for a range of neurodegenerative disorders (Menzies et al., 2017). BBR, as an autophagy modulator, affects autophagy by affecting targets or pathways associated with autophagy (AMPK, mTOR, MAPK, Beclin-1, and SIRT1) (Figure 5). Furthermore, the effect of BBR depends on the environment, and its regulation of autophagy is bidirectional, not only promoting autophagy, but also inhibiting autophagy (Mohammadinejad et al., 2019). AMPK/mTOR is a basic modulator of autophagy, and BBR can regulate AMPK/mTOR through transcriptional mechanism. BBR significantly increases AMPK activity through the production of ROS and the elimination of AMPKα1 (Fan et al., 2015; Issat et al., 2011). BBR inhibits PI3K activity, significantly downregulates AKT phosphorylation in a manner that depends on the dosage, and inhibits the phosphorylation of mTOR, p70 ribosome S6 protein kinase (Thr389) and S6 (Ser235/236), promoting the early initiation of autophagy (Huang et al., 2021; Yi et al., 2015). BBR upregulates autophagy by regulating mitogen-activated protein kinase (MAPK) (Sun et al., 2015). Beclin-1 has binding sites for Vps34 and Bcl-2. When autophagy is induced, Beclin-1 separates out from Bcl-2 and binds to Vps34, enhancing the binding force between Beclin-1 and Vps34, which inducing autophagy (Fleming et al., 2011). BBR can upregulate the expression of Beclin-1 and promote its binding to Vps34. Interestingly, BBR can also inhibit autophagy and produce neuroprotective effects by making Bcl-2 and Beclin-1 bind continuously (Ding, S. et al., 2021). SIRT1 can activate autophagy and promote the expression of genes associated with autophagy by means of FOXO. BBR can enhance the deacetylation activity of SIRT1 and induce autophagy (Lapierre et al., 2015; Sun et al., 2018a).

### 2.5 Promote angiogenesis

Angiogenesis is achieved by stimulating the growth of blood vessels, which can promote the survival of neurons, improve brain plasticity and restore nerve function (Yang and Torbey, 2020), Figure 6 shows the mechanism of BBR promoting angiogenesis. BBR activates AMPK signaling pathway and has a vital function in promoting angiogenesis. BBR also promotes alternative activation (anti-inflammatory M2) polarization of microglia and inhibits classical activation (pro-inflammatory M1) polarization through AMPK signaling pathway activation to promote angiogenesis (Zhu et al., 2019). Zhu et al. found that BBR activated Akt and subsequently increased angiogenesis by activating miR-29b expression *in vitro* and *in vivo* (Zhu et al., 2017). Vascular endothelial growth factor (VEGF) is a strong and essential pro-aging factor, which plays a crucial role in angiogenesis. Zou et al. (2019), Tian et al. (2023)’s study showed that BBR could promote the expression of VEGF and induce angiogenesis. By activating the HIF-1α/VEGF signal transduction pathway, BBR can increase the expression of microvascular density, VEGF and hypoxia inducible factor-1α (HIF-1α), promote angiogenesis, and thus achieve neuroprotective effects (Liu et al., 2017).

**FIGURE 6:**
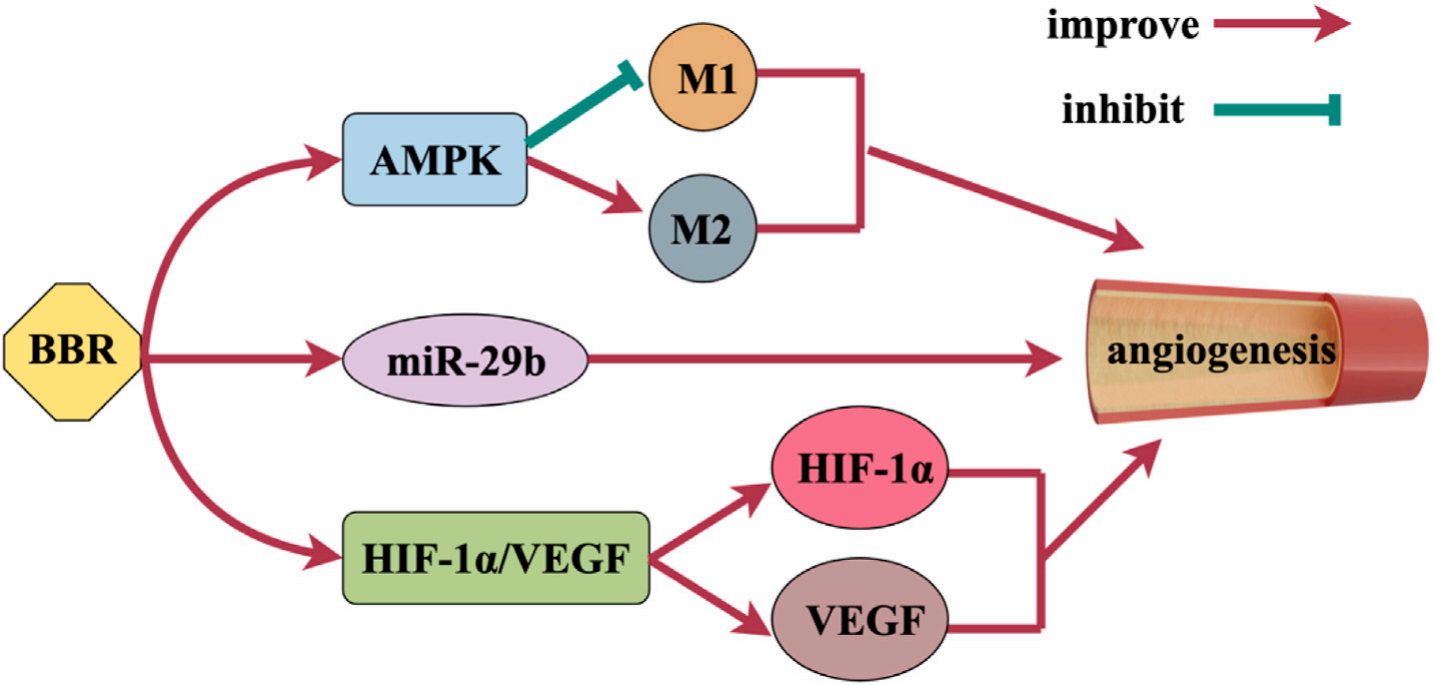
Mechanism of BBR promoting angiogenesis.

## 3 BBR and central nervous system diseases

### 3.1 Cerebrovascular diseases

#### 3.1.1 Transient cerebral ischemia, ischemia-reperfusion, stroke and cerebral infarction

**FIGURE 7:**
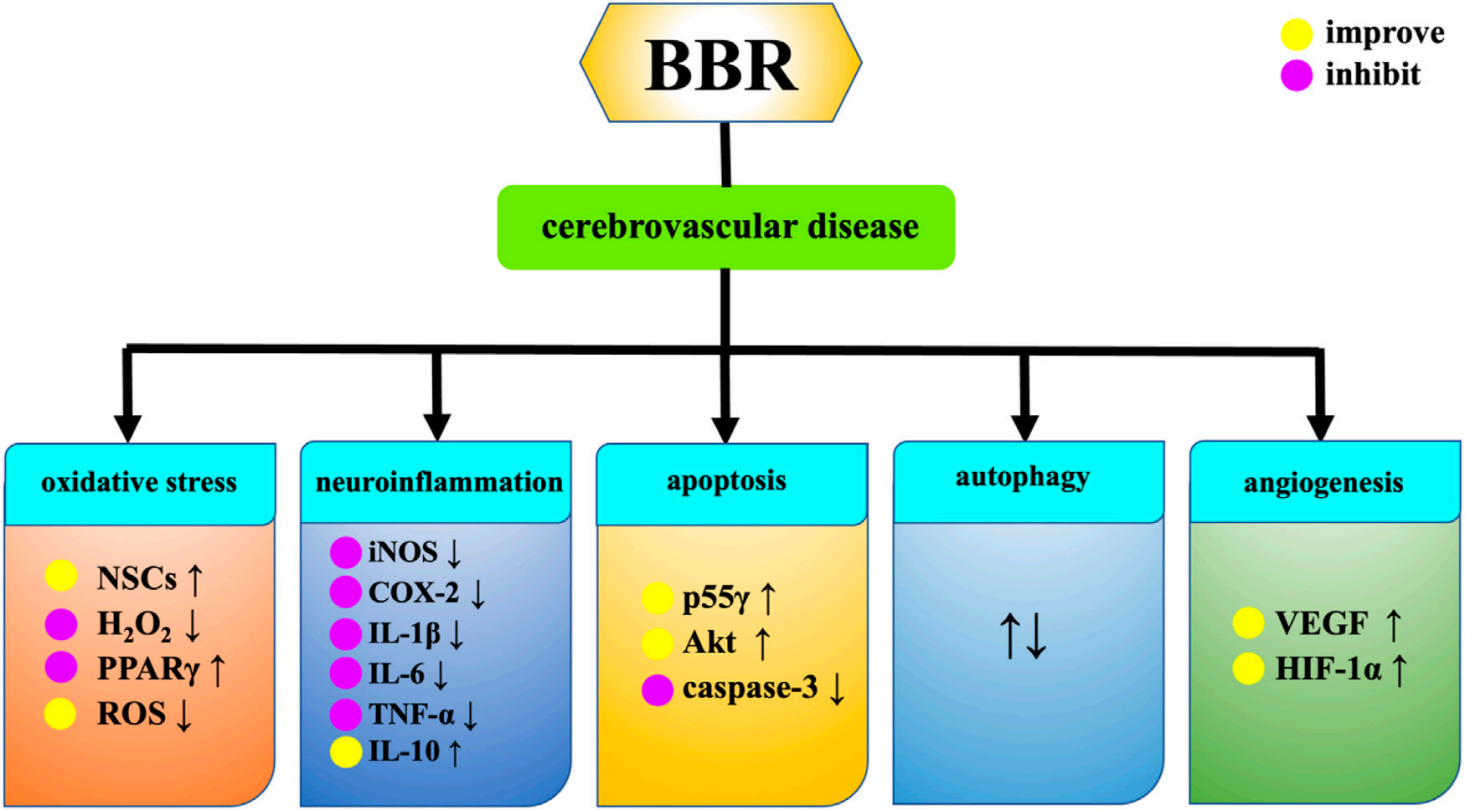
Effects of BBR on cerebrovascular diseases.

Cerebral ischemia affects all ages, from newborns to the elderly population, and is a major cause of death and morbidity (Dietz et al., 2022). Hydrogen peroxide (H_2_O_2_) is a highly active molecule in the oxidation process. The imbalance between the antioxidant system and the oxidative system is participated in neurodegeneration and ischemic brain injury. The pharmacological mechanism of BBR in treating cerebrovascular diseases is presented in Figure 7. BBR can enhance the activity of NSCs damaged by H_2_O_2_, improve the damaged morphology of cells, and improve the proliferation inhibition induced by H_2_O_2_, resulting in brain tissue reconstruction (Ye, A. L. et al., 2015). Intraperitoneal injection of BBR solution could upregulate the expression levels of phosphorylatioed protein kinase B (p-Akt), phosphorylated glycogen synthase kinase-3 (p-GSK3) and cAMP-response element binding protein (p-CREB). It also reduces the expression of nuclear factor-kappaB (NF-κB), and plays a protective role in brain tissue after cerebral ischemia (Zhang, X. L., 2013). BBR reduces the outflow of potassium ions from ischemic neurons, inhibits neuronal apoptosis in hippocampal CA1 region after ischemia, and thus protects ischemic brain tissue (Wang et al., 2004). Chai et al. discovered that BBR can specifically bind to poly(A) signal in order to control the RB1 mRNA. This prevents the degradation of RB1 mRNA and the increase in Rb protein levels during ischemia reperfusion damage, thereby regulating the release of transcription factors, blocking cell cycle, inhibiting apoptosis and promoting cell survival (Chai et al., 2014). BBR can activate the sphingosine-1-phosphate (S1P)/hypoxia inducible factor-1 (HIF-1) pathway, which is conducive to improving neuronal cell damage caused by ischemia and hypoxia (Zhang Z. et al., 2016). On the basis of the research conducted by Hu et al. (Hu et al., 2012), BBR enhances the activity of PI3K p55γ promoter during cerebral ischemia-reperfusion, resulting in increased Akt activity and decreased caspase-3 activity, thereby exerting an anti-ischemic apoptosis effect. BBR enhances the expression of peroxidase proliferator activating receptor (PPARγ) in ischemia-reperfusion injury, which may be related to the decrease of recombinant DNA methyltransferase 1 (DNMT1) and recombinant DNA methyltransferase 3A (DNMT3a) expression and the decrease of PPARγ promoter methylation in ischemia-reperfusion injury (Pang et al., 2018). In addition, BBR can also achieve neuroprotective effect by inhibiting inflammatory response. The study of Yoo et al. found that the protective effect of BBR on the brain of ischemia-reperfusion injured gerbils is also related to the inhibition of COX-2 expression, prostaglandin E2 generation and its anti-inflammatory mechanism (Yoo et al., 2008). BBR can reduce the neuroinflammatory response by down-regulating the expression of metastasis-associated lung adenocarcinoma transcript 1 (Malat1) and high mobility group box 1 (HMGB1). It protects neuronal cells from cerebral ischemia-reperfusion injury (Cao et al., 2020). BBR downregulates the levels of proinflammatory cytokines iNOS, COX-2, IL-1β, IL-6 and TNF-α, and upregulates the expression of anti-inflammatory cytokine IL-10 through targeting MAPK pathway and AMPK independent mode, alleviates the inflammatory response caused by ischemia reperfusion in rats, exerts the neuroprotective effect in brain. In addition, BBR can also carry the exosome miR-182-5p to injured neurons and play a neuroprotective role by inhibiting neuroinflammation and improving brain injury after ischemic stroke (Ding et al., 2023). Moreover, BBR inhibits ischemic neuronal death by reducing the activity of type 1 N-methyl-D-aspartate receptor (NMDA), reducing the excessive excitatory amino acids produced by ischemic stimulation and Ca^2+^ inflow caused by oxygen free radicals (Yoo et al., 2006). BBR also inhibits central sympathetic nerves by blocking α-adrenergic receptors, affecting cerebral blood flow supply in stroke patients (Benaissa et al., 2009). Autophagy is an important catabolic process in lysozyme and an essential pathway for survival in ischemic stroke (Li, H. Y. et al., 2024). For cerebral infarction, BBR can effectively reduce blood lipid levels, inhibit the expression of oxidized low-density lipoprotein (ox-LDL) and matrix metalloproteinase-9 (MMP-9), inhibit the growth of carotid atherosclerotic plaque, reduce plaque area, improve plaque stability, and improve the long-term neurological score of cerebral infarction patients (Chai, M. J. et al., 2017a). BBR can increase the level of serum catalase (CAT) and reduce the expression of Malondialdehyde (MDA) in patients with acute cerebral infarction, thereby improving the neurological deficits (Chai, M. J. et al., 2017b). BBR reduce the serum IgG level of patients with acute cerebral infarction, which may inhibit the humoral immune response in the acute phase of cerebral infarction. BBR alleviates inflammatory response in rats with acute cerebral infarction, and its mechanism may be related to activation of Wnt/β-catenin signaling pathway (Yao, Y. et al., 2023). Xi et al. suggested that BBR can protect ischemic neurons by reducing the content of E-Selectin and intercellular call adhesion molecule-1 (ICAM-1) in the plasma of patients with cerebral infarction (Xi, G. M. et al., 2003). Additionally, BBR can also reduce ischemia-reperfusion induced cerebral infarction (Zhou et al., 2008).

#### 3.1.2 Cerebrovascular disease in patients with diabetes and hyperhomocysteinemia

Diabetic peripheral neuropathy (DPN) is the most common chronic complication of diabetes mellitus (DM). Hyperhomocysteinemia (HHcy) can also lead to DPN. BBR improves nerve conduction velocity in diabetic peripheral neuropathy patients. The combination of mecobalamine with DPN can improve the symptoms and nerve conduction velocity of DPN patients, and has significant clinical efficacy (Ye, A. L. et al., 2015). Zhao et al. found through their research (Zhao, X. L. et al., 2006) that BBR can reduce the damage of brain cells, improve the compensatory function of brain cells and reduce blood sugar by alleviating mitochondrial calcium overload and release mitochondrial Cyt-C, thus playing a protective role in the brain tissue of diabetic rats.

### 3.2 Multiple sclerosis

Multiple sclerosis (MS) is an autoimmune disease characterized by immune imbalance, central nervous system inflammatory response, and myelin destruction. Experimental autoimmune encephalomyelitis (EAE) is the recognized animal model of this disease (Kroenke et al., 2008). In the inflammatory response of EAE, Th1 cells and Th17 cells are activated, and the secretion of inflammatory cytokines (INF-γ, IL-6, IL-17) respectively increases. BBR can directly act on the JAK/STAT signaling pathway and selectively inhibit the differentiation of Th1 and Th17 cells. BBR indirectly affects the function of Th1 and Th17 cells by affecting the expression and function of co-stimulatory molecules and the production of IL-6, which is due to inhibition of NF-κB activity in CD11b (+) APC (Qin et al., 2010). In addition, BBR can also inhibit the expression and activity of MMP-9, inhibit the migration of T cells to the central nervous system, and protect the blood-brain barrier, thereby reducing the inflammatory infiltration of the central nervous system, alleviating the disease of EAE mice, slowing down the inflammatory response and reducing the incidence of demyelination (Ma et al., 2010).

### 3.3 Glioma

Due to its invasive nature, molecular signaling, and location in the central nervous system, glioma is one of the most perplexing cancers (Kundu et al., 2019). BBR-mediated apoptosis blocks the AMPK/mTOR/ULK1 pathway and reduces tumor growth in glioblastoma multiforme (GBM) cells *in vivo* (Wang et al., 2016). BBR inhibits tumor growth and inhibits the expression of p-ERK1/2 and Ki-67 in glioma cells (Sun et al., 2018b). Neuroinflammatory cytokines such as IL-1 secreted by glioma cells are believed to have an impact on the genesis and development of tumors (Nasrollahzadeh et al., 2020). BBR inhibits the activation of inflammatory cytokine caspase-1 through the ERK1/2 signaling pathway, inhibits glioma cells, and subsequently produces IL-1 and IL-18. BBR has a specific anti-proliferation effect on glioma cells, and treatment with BBR can also reduce the cellular motility of U251 and U87 cells and induce cell apoptosis. BBR induces apoptosis of T98G cells in glioblastoma by mediating endoplasmic reticulum stress (Eom et al., 2010). BBR inhibits TGF-β1/SMAD2/3 signaling pathway and affects the proliferation, migration, invasion and apoptosis of glioma cells (Jin et al., 2022). Moreover, BBR has the potential to reverse the mechanism of epithelial-mesenchymal metastasis, which is a hallmark of tumor invasion (Tong et al., 2019). BBR inhibits tumor development by regulating neuroblastoma cell differentiation, stem cell function, and inducing cell death (Rauf et al., 2021).

### 3.4 Epilepsy

Epilepsy is one of the most widespread neurological diseases in the world. According to the study of Ghanem et al. (Ghanem et al., 2021), BBR can significantly reduce the activity of hypoxia inducible factor-1α (HIF-1α), transforming growth factor-β1 (TGF-β1), histone deacetylase (HDAC) and neuronal restrictive silencing factor NRSF gene expression levels in epileptic mice, and increase the level of Brain-derived neurotrophic factor (BDNF). Intraperitoneal injection of BBR in the mouse model of maximal electroshock-induced seizures can reduce the duration of the seizure of the tonic hind limb extension, resist the seizure of convulsions, and reduce the mortality (Bhutada et al., 2010). The study of Zhang et al. showed that BBR can alleviate pentylenetetrazole (PTZ)-induced seizures, potentially protect zebrafish from further seizures, and use BBR can restore abnormal neuron firing in zebrafish larvae during seizures. In addition, BBR also suppresses the inflammatory response caused by epilepsy (Zhang et al., 2020). Sedaghat et al. found that BBR prevents the loss of hippocampal CA3 neurons and prevents the development of abnormal mossy fiber sprouting (MFS), which is a basic element of the action circuit in chronic epilepsy (Sedaghat et al., 2017). These data suggest that BBR also exerts a neuroprotective effect by alleviating Status epilepticus (SE) and spontaneous recurrent seizures (SRS) in the hippocampal model of epilepsy.

### 3.5 Neurodegenerative diseases

**FIGURE 8:**
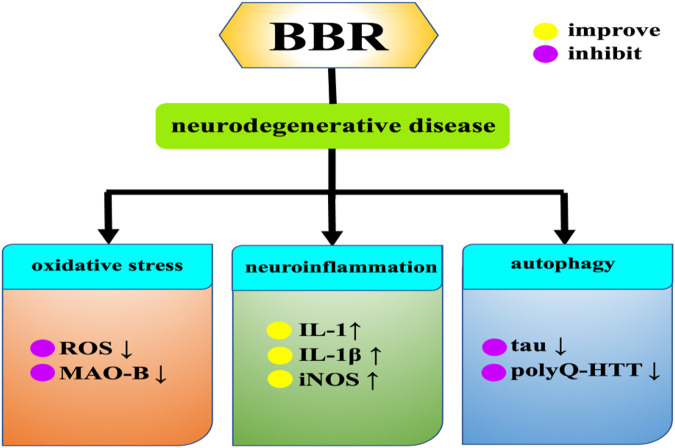
Effects of BBR on neurodegenerative diseases.

With the increase of the elderly population, age-related diseases such as neurodegenerative diseases are becoming more and more common, posing a threat to human health (Heemels, 2016). Specifically, neurodegenerative diseases include Alzheimer’s disease, Parkinson’s disease, Huntington’s syndrome, etc. The pathogenesis of neurodegenerative diseases has several common features, such as oxidative damage and mitochondrial dysfunction. Among them, oxidative stress refers to excessive accumulation of ROS, which can lead to mitochondrial dysfunction and ultimately induce neurodegenerative diseases. BBR significantly reduces ROS production in cytoplasm and mitochondria (Caspersen et al., 2005; Poprac et al., 2017), Moreover, BBR also alleviates neurodegenerative diseases by regulating neuroinflammation and autophagy **(** Figure 8).

#### 3.5.1 Alzheimer’s disease

Alzheimer’s disease (AD) is a neurological illness characterized by memory loss and cognitive impairment, accounting for around 60%–80% of all dementia cases (Rostagno, 2022). Aβ deposition is a crucial factor in the development of Alzheimer’s disease (Huat et al., 2019). BBR can improve the effect of AD through various mechanisms, including inhibiting the hyperphosphorylation of Tau protein, inhibiting the production of Aβ, and inhibiting four key enzymes (acetylcholinesterase, butylcholinesterase and two isomers of monoamine oxidase) in the pathogenesis of AD. BBR can reduce the phosphorylation level of Tau protein, which may be related to its activation of the Akt/glycogen synthase kinase three signaling pathway (Durairajan et al., 2012). BBR can reverse the increase of malondialdehyde content and the decrease of SOD activity induced by calyculin A. Calyculin A-induced tau hyperphosphorylation is decreased by increasing protein phosphatase 2A (PP2A) activity and decreasing glycogen synthase kinase 3β (GSK-3β) activity (Yu et al., 2011). BBR can also enhance autophagy activity and promote autophagy clearance of tau through Class III PI3K/beclin-1 pathway (Chen et al., 2020). BBR significantly reduces Aβ, possibly by down-regulating the phosphorylation of amyloid precursor protein (APP) through activation of PI3K/Akt/GSK3 pathway, and inhibiting the production of Aβ_42_ by inhibiting PERK/eIF2α/BACE1 signaling pathway (Wu et al., 2021). BBR can also promote Aβ clearance by promoting the expression of insulin-degrading enzyme (IDE) (Zhu, F. Q. et al., 2010). Inhibition of AChE and BChE has been shown to be a key target for effective management of AD by alleviating cholinergic deficiency and improving neurotransmission, and BBR has inhibitory effects on both AChE and BChE (Greig et al., 2005; Muñoz-Torrero, 2008). Not only that, BBR can also activate macrophages and increase their phagocytic function, increase the production of interleukin, (IL)-1 and can be used as a neuroprotective agent against AD (Kumazawa et al., 1984; Panahi et al., 2013). Long-term administration of BBR increases the expression of IL-1β and iNOS in the hippocampus of AD mice and improves memory impairment (Ji and Shen, 2012).

#### 3.5.2 Parkinson’s disease

Parkinson’s disease (PD) is the second most common neurological disorder, affecting about seven million people worldwide (Pringsheim et al., 2014). Inhibition of Monoamine oxidase-B (MAO-B) has been proved to delay the onset of PD or reduce the symptoms of PD (Youdim et al., 2006). BBR and MAO-B are bound by hydrophobic interaction, and BBR may partially reduce the degradation of dopamine and the production of H_2_O_2_ through the inhibition of MAO-B, thus alleviating the symptoms of PD. Kim et al. (Kim et al., 2014a) used 1-methyl-4-phenyl-1,2,3,6-tetrahydropyridine/probenecid (MPTP/P) mouse PD model to study the effects of BBR on dopamine consumption and short-term memory of hippocampal neurogenesis. BBR was found to enhance motor balance and coordination by preventing damage to dopaminergic neurons, also improved short-term memory by inhibiting apoptosis of hippocampal cells. These data show that BBR treatment may serve as a potential therapeutic strategy to improve memory impairment and physical dysfunction in PD patients. In the study of Negahdar et al. (Negahdar et al., 2015) by using 6-hydroxydopamine (6-OHDA) -induced PD model, BBR could significantly improve lateral rotation behavior in PD rats, and could also prevent the reduction of the number of tyrosine-hydroxylase (TH) -positive neurons in the group, providing a new strategy for PD treatment. It is worth noting that BBR can enhance the cytotoxicity caused by 6-OHDA, and the intraparitoneal injection of BBR for 21d in the 6-OHDA PD rat model can cause degeneration of dopaminergic neurons in the substrantia nigra (Kwon et al., 2010). Therefore, patients with PD should pay attention to the interaction between BBR and levodopa and avoid drug effects when using BBR.

#### 3.5.3 Huntington’s disease

Huntington’s disease (HD) is an inherited neurodegenerative genetic disorder caused by the amplification (variable length) of CAG trinucleotide repeats in HTT, the gene encoding the Huntington protein, which accumulate in affected brain regions in an age-dependent manner, leading to late-onset neurodegeneration (Walker, 2007). In Jiang et al.’s study (Jiang W. et al., 2015), BBR has a protective effect on transgenic HD (N171-82Q) mice. When taken orally, BBR can effectively alleviate motor dysfunction and prolong the survival time of transgenic HD mice, and BBR can also promote the degradation of mutant Huntington protein by enhancing autophagy function. The autophagy-lysosome pathway also plays a crucial role in the clearance of the readily aggregated mutant Huntington protein (polyQ-HTT) (Martinez-Vicente et al., 2010), and induction of autophagy enhances the clearance of polyQ-HTT aggregates and reduces the toxicity of the mutant Huntington protein fragment (Floto et al., 2007). BBR can significantly trigger autophagy and remove polyQ-HTT aggregates, thereby significantly improving the neurophenotype of HD mice (Jiang W. et al., 2015; Fan et al., 2019).

### 3.6 Mental system disorders

A mental illness is a disorder of brain function caused by a variety of reasons that manifests in different forms as disorders in mental functions like cognition, behavior, will and emotion.

#### 3.6.1 Anxiety

Anxiety is a state of excessive fear, characterized by motor tension, sympathetic overactivity, worry, and vigilance syndrome. BBR plays an anti-anxiety role by reducing the concentration of norepinephrine, dopamine and 5-hydroxytryptamine in the brain stem, and increasing the concentration of Vanillymandelic Acid (VMA) and 4-hydroxy-3-methoxy-phenylacetic acid (HVA). The anti-anxiety effect of 100 mg/kg BBR on mice is the same as that of 1 mg/kg diazepam and 2 mg/kg buspirone, and its anti-anxiety effect is related to accelerating the renewal rate of monoamine transmitters in the brain stem and reducing the activity of 5-HT-ergic systems (Peng et al., 2004). Yu et al. found that BBR improved anxiety in 5XFAD transgenic mice with AD (Yu, W. et al., 2021). Autonomic nervous system dysfunction and anxiety and other mental disorders often occur in women before and after menopause due to the fluctuation or reduction of sex hormone levels (Mulhall et al., 2018). BBR can also increase the content of equol in feces and serum and the ratio of equol to daidzein by enriching *Lactobacillus*, *Bacteroides*, *Bifidobacterium* and *Akkermansia muciniphila* in the intestine. Improved anxiety-like behavior in female ovariectomized rats. The regulatory effect of BBR was eliminated in germ-free animals, but the changes in microbiota, equol content and anxiety-like behavior of the animals receiving fecal microbiota transplantation were basically the same as those of the donors. This suggests that BBR can improve anxiety-like behaviors induced by decreased ovarian hormones by regulating intestinal microbiota and promoting equol conversion (Fang, Y., 2022; Fang et al., 2021).

#### 3.6.2 Depression

Depression is a relatively common disease with the highest suicide rate in psychiatric departments, mainly manifested by low mood, anxiety, insomnia, loss of appetite, and inconcentration (Malhi and Mann, 2018). Norepinephrine, serotonin, and dopamine are substrates for monoamine oxidase (MAO), and MAO inhibitors have antidepressant activity, BBR may regulate the levels of brain biogenic amines (norepinephrine, serotonin, and dopamine) by interacting with adrenaline receptors, 5-HT, dopamine, and MAO, and exert antidepressant-like effects in various depression models. Moreover, the antidepressant like effect of BBR in Forced Swimming Test (FST) involves interaction with the L-arginine-NO-cGMP pathway (Peng et al., 2007; Kulkarni and Dhir, 2008; Kulkarni and Dhir, 2007). Organic cation transporters (OCTs) and plasma membrane monoamine transporters (PMAT) are the most efficient transporters for uptake of 5-HT, NE and other biogenic amine neurotransmitters (Daws et al., 2013). Sun et al.'s study found that BBR can play an antidepressant role by inhibiting OCT2 and OCT3 (Sun et al., 2014). The study of Yi et al. showed that BBR also exerts antidepressant effects by regulating the miR-34a-synaptotagmin1/Bcl-2 axis (Yi et al., 2021). BBR may also inhibit NF-κB signaling pathway and its downstream targets such as proinflammatory cytokines and iNOS exert antidepressant effects (Liu et al., 2017). The HPA axis is an important part of the neuroendocrine system and is closely related to depression. The activation of HPA axis is manifested by increased secretion of corticotrophin-releasing factor (CRF) in the hypothalamus. Then the pituitary adrenocorticotrophin (ACTH) release. BDNF has been shown to be a key contributor to antidepressant effects (Erickson et al., 2012), and excessive plasma corticosterone (CORT) is also an important trigger for depressive episodes (Gong et al., 2019). BBR combined with ginsenoside can upregulate the expression level of plasma BDNF and downregulate the levels of CORT and ACTH (Shen et al., 2016; Zhang et al., 2021). BBR can also significantly reduce the expression of CRF in hypothalamus, and significantly improve the depressive behavior of rats with chronic morphine withdrawal (Lee et al., 2012; Gao et al., 2024).

#### 3.6.3 Schizophrenia

Schizophrenia is a chronic and severe mental illness in which dopamine-mediated neurotransmission plays a crucial role in psychiatric and nervous system disorders. The prolyl oligopeptidase (POP) family of enzymes are cytoplasmic serine peptidases, and the activity of POP is reduced in depression and increased in psychiatric disorders such as mania and schizophrenia (Maes et al., 1995). BBR inhibits POP in a concentration dependent manner, thereby exerting anti schizophrenia effects (Tarrago et al., 2007). TetrahydroprotoBBRs (THPBs), a derivative of BBR, separated from Chinese herbal medicine compound, through its unique D2 receptor antagonist and agonist activity of D1, play a role of resistant schizophrenia (Chu et al., 2008; Kulkarni and Dhir, 2010). Ghotbi et al. created a rat schizophrenia model by administering MK-801 (NMDA receptor antagonist) (Ghotbi Ravandi et al., 2019), and the results showed that BBR had neuroprotective effects on rats with MK-801-related behavioral defects, suggesting that BBR had anti-schizophrenic effects (Shayganfard, 2023).

## 4 Conclusion

**FIGURE 9:**
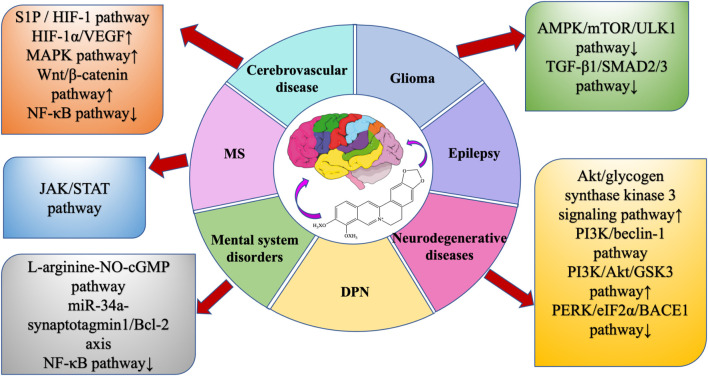
Neuroprotective pathway of BBR.

**FIGURE 10:**
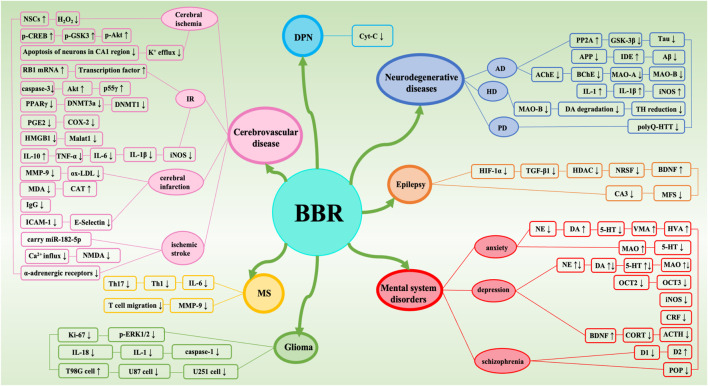
Target of neuroprotective effect of BBR.

BBR is widely present in the roots, rhizomes, stems, or bark of many traditionally used herbs, and has a wide range of physiological activities, especially in neuroprotection, but its bioavailability is low, so the study of BBR dosage forms is very important to improve bioavailability. This review summarizes the extraction methods, dosage forms, pharmacological effects, and protective effects of BBR on the central nervous system, and summarizes a large number of studies, indicating that BBR can directly or indirectly regulate various intracellular molecules and signaling pathways (Figures 9, 10) , thereby improving nervous system diseases. Such as cerebrovascular disease, multiple sclerosis, glioma, epilepsy, AD, PD, HD, anxiety, depression, and schizophrenia.

At present, the complex network mechanism of BBR is not fully understood, which may be due to the interaction with multiple targets or with proteins that are involved in many pathways. In order to further study the pharmacological mechanism of berberine, it can be revealed by exploring the possible protein targets directly acting on berberine (some researches on the targets of direct BBR action are shown in Table 3). Researches on the targets of direct BBR action can not only reveal the mechanism of therapeutic effect of BBR from a fundamental perspective, but also provide new strategies for the design of drug combinations. However, there are few studies on the direct targets of BBR for the treatment of neurological diseases, so it is important to further explore BBR, which will help clarify the multi-active mechanism of BBR’s neuroprotective effect and its corresponding biological effects.

**TABLE 3:** Targets of direct BBR action.

Direct target of berberine	Disease	Technology	Significance	Reference
EIF2AK2, eEF1A1, PRDX3, and VPS4B	Inflammatory	ABPP	It may be an important therapeutic target for inflammation-related diseases	Wei et al. (2023)
NEK7 protein	Inflammatory	ABPP	New inhibitors of NEK7-NLRP3 interaction may be developed	Zeng et al. (2021a)
FtfL	Colorectal cancer	ABPP	Dissect related mechanisms that prevent the occurrence and development of colorectal cancer from the perspective of intestinal microorganisms	Yan et al. (2023)
RXRα	Colorectal cancer	Luciferase assay, Lentiviral vector-based shRNA technique, Isothermal titration calorimetry, etc	Develop new strategies for designing a new RXRα-based anticancer agents and medication combinations	(H et al., 2017)
UHRF1	Multiple myeloma	SPR, LC-MS/MS	It is helpful for the treatment of multiple myeloma by BBR	Gu et al. (2020)
MAP2K7	Obesity, Neurodegenerative diseasesetc.	ABPP, CETSA	Propose MAP2K7 as a druggable target for the development of selective JNK pathway modulators	Zeng et al. (2021b)
Actin	Cancer	An affinity-based chemical probe of berberine, Mass spectrometry	It provides a reasonable explanation that BBR inhibits cell migration and cancer cell invasion	Yi et al. (2017)

## Figures and Tables

**Table:** 

**5-HT**	5-hydroxytryptamine
**6-OHDA**	6-hydroxydopamine
**ABPP**	Activity-based protein profiling
**AChE**	Acetylcholinesterase
**ACTH**	Adrenocorticotrophin
**AD**	Alzheimer’s disease
**ADP**	Adenosine diphosphate
**Akt**	Protein kinase B
**AMP**	Adenosine monophosphate
**AMPK**	(AMP)-activated protein kinase
**APP**	Amyloid precursor protein
**BACE1**	Beta-Site APP Cleaving Enzyme 1
**Bax**	Bcl-2 associated X
**BBR**	BBR
**BChE**	Butylcholinesterase
**Bcl-2**	B-cell lymphoma 2
**BDNF**	Brain-derived neurotrophic factor
**CAT**	Catalase
**CETSA**	Cellular thermal shift assay
**CMC-Na**	Carboxymethylcellulose sodium
**CORT**	Corticosterone
**COX-2**	Cyclooxygenase-2
**CREB**	Cyclic AMP Response Element-binding Protein
**CRF**	Corticotrophin-releasing factor
**Cyt-C**	Cytochrome C
**DM**	Diabetes mellitus
**DNMT1**	DNA methyltransferase 1
**DNMT3a**	DNA methyltransferase 3a
**DPN**	Diabetic peripheral neuropathy
**EAE**	Experimental autoimmune encephalomyelitis
**EIF2AK2**	Initiation factor 2 alpha kinase 2
**eEF1A1**	Elongation factor 1 alpha 1
**eIF2α**	Eukaryotic Initiation Factor 2α
**ERK**	Extracellular regulated protein kinases
**FOXO1**	Forkhead box O1
**FtfL**	Formate tetrahydrofolate ligase
**GBM**	Glioblastoma multiforme
**GFAP**	Glial fibrillary acidic protein
**GSH**	Glutathione
**GSK3β**	Glycogen synthase kinase-3β
**H**_**2**_ **O**_**2**_	Hydrogen peroxide
**HD**	Huntington’s disease
**HDAC**	Histone deacetylase
**HHcy**	Hyperhomocysteinemia
**HIF-1**	Hypoxia inducible factor-1
**HIF-1α**	Hypoxia-inducible factor-1α
**HMGB1**	High mobility group box 1
**HO-1**	Heme oxygenase 1
**HPA**	Hypothalamic-pituitary-adrenal
**HPMCP**	Hydroxypropyl methylcellulose phthalate
**HVA**	4-hydroxy-3-methoxy-phenylacetic acid
**ICAM-1**	intercellular cell adhesion molecule-1
**IDE**	Insulin-degrading enzyme
**IFN**	Interferon
**IL-1**	Interleukin-1
**IL-6**	Interleukin-6
**IL-10**	Interleukin-10
**IL-17**	Interleukin-17
**IL-18**	Interleukin-18
**IL-1β**	Interleukin-1β
**INF-γ**	Interferon-γ
**iNOS**	Inducible nitric oxide synthase
**JAK**	Janus Kinase
**JNK**	c-Jun N-terminal kinase
**LC3**	Microtubule-associated protein 1A/1B-light chain 3
**LC-MS/MS**	Liquid chromatography-tandem mass spectrometry
**LPS**	Lipopolysaccharide
**M1**	Berberrubine
**M2**	Thalifendine
**M3**	Demethyleneberberine
**M4**	Jatrorrhizine
**Malat1**	Metastasis-associated lung adenocarcinoma transcript 1
**MAO-A**	Monoamine oxidase-A
**MAO-B**	Monoamine oxidase-B
**MAP2K7**	Mitogen-Activated Protein Kinase Kinase 7
**MAPK**	Mitogen-activated protein kinase
**MDA**	Malondialdehyde
**MFS**	Mossy fiber sprouting
**MiR-29b**	MicroRNA-29b
**MMP-9**	Matrix metalloproteinase-9
**MPTP/P**	1-methyl-4-phenyl-1,2,3,6-tetrahydropyridine/probenecid
**MS**	Multiple sclerosis
**mTOR**	Mammalian target of rapamycin
**NADPH**	Nicotinamide adenine dinucleotide phosphate
**NE**	Norepinephrine
**NEK7**	NIMA-related kinase 7
**NF-κB**	Nuclear factor-k-gene binding
**NMDA**	N-methyl-D-aspartate
**NRSF**	Neuronal restrictive silencing factor
**NSCs**	Neural Stem Cells
**OCTs**	Organic cation transporters
**ox-LDL**	Oxidized low-density lipoprotein
**p70S6K**	p70 ribosome S6 protein kinase
**p-Akt**	Phosphorylatio protein kinase B
**PARP**	Poly ADP-ribose polymerase
**p-CREB**	cAMP-response element binding protein
**PD**	Parkinson’s disease
**PEG**	Polyethylene glycol
**PERK(p-ERK)**	Phosphorylated extracellular regulated protein kinases
**p-ERK1/2**	Phosphorylated extracellular regulated protein kinases1/2
**P-gP**	P-glycoprotein
**p-GSK3**	Phosphorylated glycogen synthase kinase-3
**PI3K**	Phosphatidylinositol-3-kinase
**PMAT**	Plasma membrane monoamine transporters
**Poly(A)**	Polyadenylic acid
**PolyQ-HTT**	Polyglutamine-Huntington
**POP**	Prolyl oligopeptidase
**PP2A**	Phosphatase 2A
**PPAR**	Peroxisome proliferator-activated receptor
**PRDX3**	Peroxiredoxin 3
**PTZ**	Pentylenetetrazole
**PVA**	Polyvinyl alcohol
**RB**	Retinoblastoma
**RB1 mRNA**	Retinoblastoma 1 mRNA
**RNS**	Reactive nitrogen species
**ROS**	Reactive oxygen species
**RXRα**	Retinoid X receptor alpha
**S1P**	Sphingosine-1-phosphate
**SE**	Status epilepticus
**SIRT1**	Sirtuin1
**SMAD**	(Sma-Mad) family
**SOD**	Superoxide dismutase
**SPR**	Surface plasmon resonance
**Sphk1**	Sphingosin kinase 1
**SRS**	Spontaneous recurrent seizures
**STAT**	Signal transducer and activator of transcription
**TGF-β1**	Transforming growth factor-β1
**TH**	Tyrosine-hydroxylase
**THPBs**	TetrahydroprotoBBRs
**TNF-α**	Tumor necrosis factor-α
**UHRF1**	Ubiquitin-like with PHD and RING Finger domains 1
**ULK1**	Vascular endothelial growth factor
**VEGF**	Vascular endothelial growth factor
**VMA**	Vanillymandelic Acid
**VPS4B**	Vacuolar protein sorting 4 homolog B
**Wnt**	Wingless/Integrated
