# Supplementary material for: Neuroprotective effect and preparation methods of berberine
Source: Front Pharmacol. 2024 Sep 6;15:1429050. doi: 10.3389/fphar.2024.1429050 (PMC11412855; doi:10.3389/fphar.2024.1429050)
Supplement: Supplementary file 1 [file DataSheet1.docx]

Supplementary Material

# Supplementary Tables and Figures

## Supplementary Tables

**Supplementary Table 1.** Different extraction methods of BBR

| Method | | Procedure | Characteristic | Reference |
| --- | --- | --- | --- | --- |
| Acid water extraction | | The raw materials are soaked in a multiple amount of sulfuric acid water for 24h, the PH of the filtrate is adjusted to 10 ~ 12 with lime milk, filtration, the PH of the filtrate is 2 ~ 3 controlled by hydrochloric acid solution, and the refined salt is added, completely dissolved, placed overnight, suction filtration to obtain crude product. | The extraction method is simple and low cost, But the extraction rate is low, unsafe, not environmentally friendly, easy to corrode equipment. | (Liao, Z. X. et al., 1998; Pang, X. X. and Xu, W. L., 1996) |
| Alkali water extraction | | Add the raw materials into the lime milk, stir evenly, soak in saturated lime water for 6h, percolation, control the flow rate, add solid salt into the percolate, filtration, precipitate, dissolve in hot water, filter while its hot, add hydrochloric acid to adjust the PH to 2, stewing, filtration, wash the precipitate with water until neutral, suction filtration to obtain crude product. | Compared with acid water extraction and alcohol extraction, the extraction efficiency is relatively better.However, due to the extensive use of lime milk, it may cause partial component loss. | (Cheng, Y. M. and Chen, R. H., 2007; Yin, R. L. et al., 2000) |
| Alcohol extraction | Microwave-assisted extraction | Soak the raw material powder with ethanol, recover most of it, concentrate the rest, filtration, salt-out acid extraction, precipitation, stewing, obtain crude product. | Fast heating, uniform heating, easy to operate. But the research of extraction technology is still in the initial stage, and the parameters in the extraction process, such as the physical properties of medicinal materials, comminution degree, the content of free water or bound water, all have an impact on the extraction rate. | (Yang, Q. Z. et al., 2015; Deng, Y. H. et al., 2002) |
|  | Flash extraction | Same as above | The extraction speed is fast, suitable for most solvents, high efficiency and energy saving, and easy to operate. Due to the small amount of herbs added at one time, it will be difficult to filter due to excessive water-soluble components, increased viscosity, suspension and emulsification. | (Liu, Y. Z., 2007; Qin, Z. F. and Li, H. Y., 2005) |
|  | Ultrasonic extraction | Take raw material powder, add sulfuric acid solution, soak for 24h, ultrasonic treatment for a period of time, filtration. Take the filtrate, dilute it with water and shake well. | Ultrasonic extraction technology can use the strong vibration generated by ultrasonic wave to accelerate the active ingredients into the solvent, so as to improve the extraction rate and avoid the influence of high temperature on the extracted ingredients. But it increases the difficulty of equipment investment and operation. | (Guo, X.W. et al., 1995; Wu, B. H., 2004; Ong et al., 2000) |
| Enzymatic extraction | | The raw materials were pretreated by adding the enzyme solution, adding lime water, ultrasound, filtration, adding hydrochloric acid to adjust the PH to 2 ~ 3, adding salt and placing overnight, suction filtration to obtain the product. | The extraction temperature of enzyme reaction is low, which can significantly increase the yield. | (Liang, B. L. and Zhou, M. J., 2006) |
| Microextraction | | - | Less consumption, less funds, less environmental pollution, safety, short time;  But compared with the conventional chemical experiment, the yield is lower. | (Cheng, Y. M. and Chen, R. H., 2007) |
| Semi-bionic extraction | | Take the raw materials and add water in a certain proportion and decoct for 3 times, take PH=5.5 as the first decoction, take PH=10 as the second and third decoction, repeat twice, combine the decoction, filtration, concentrate, add talc powder, stand, centrifuge, constant volume, and get the product. | More active ingredients can be extracted and retained, shortening the production cycle and reducing costs. | (Lin, H. B. et al., 2004; Sun, X. M. et al., 1996; Zhang, Z. W. and Sun, X. M., 1995) |
| Supercritical CO_2_ extraction | | The supercritical fluid is contacted with BBR to dissolve it fully, and then the supercritical fluid CO_2_ is changed into a gas by reducing pressure and heating up, and the BBR is precipitated. | Compared with the traditional solvent extraction method, it has the advantages of low temperature, high speed, high efficiency, good pharmacological effect and lower toxicity. | (Liu et al., 2006) |
| Aqueous two phase extraction | | The crude extract of raw materials was taken and added into (NH_4_)_2_SO_4_/PEG400 two-phase aqueous solution, centrifuged into two phases, and read the volume of the two phases, and the BBR concentration in the two phases was calculated to obtain its extraction rate. | The extraction conditions are mild, the extraction phase does not contain polymer with large viscosity, and the phase separation is clearer and faster. The traditional two-phase aqueous system is generally polyethylene glycol - glucan, polyethylene glycol - inorganic salt, etc., most of them have large viscosity, hard to volatilize, and subsequent separation is more complicated. | (Yang, Q. Z. et al., 2015; Xie, T. et al., 2008; Wen,C. F. et al., 2011; Li, M. Q. et al., 2006) |
| High pressure hot water extraction | | The raw material is reflow with ethanol, filtration, concentrated and fixed volume, treated with high pressure hot water, and precipitated by recrystallization after standing and filtering. | More simple than pressurized fluid extraction, the extraction rate is high, in the appropriate pressure range, as the pressure rises, the extracted component content increases. | (Ong and Len, 2003) |
| Liquid-membrane extraction | | Pour the liquid film into the mother liquor, stir at a slow speed for 10 minutes, separate the liquid film layer that has absorbed BBR with the separator funnel, pour into the beaker, repeat twice. After that, the film is broken in the constant temperature water bath, standing, filtering, constant volume and sampling. | Improve the separation and concentration effect, without a lot of pretreatment, easy to achieve industrialization, low energy consumption, less chemical consumption, no secondary pollution, better economic benefits. | (Wang, D. J. et al., 2006) |

**Supplementary Table 2.** Different dosage forms of BBR

| **Dosage form** | **Definition** | **Procedure** | **Characteristic** | **Reference** |
| --- | --- | --- | --- | --- |
| Liposome | The superminiature spherical carrier preparation is prepared by encapsulating the drug in the middle of the thin film formed by the lipid double molecular layer | The ratio of phospholipid to cholesterol was 3∶1, the ratio of drug to lipid was 1∶15, the mass concentration of phospholipid was 30g/L, and the pH of external aqueous phase was 7.0. | It has remarkable sustained release properties in vitro. | (Wang, X. H. et al., 2013) |
|  |  | The liposomes were stable and reliable when the ratio of BBR hydrochloride to polysorbate 80 was 1∶0.4 and the ratio of soybean lipid (soybean phosphate) was 1∶20. | Targeted, long-acting, low-toxicity, slow-release, non-immunogenic and protective encapsulation drugs, can increase gastrointestinal absorption. | (Xu, L. J. et al., 2004) |
|  |  | The liposomes prepared by injection method had uniform size, average particle size of 0.79μm, high encapsulation rate, high purity, and simple and easy content detection. | Easy to operate,the synthesized BBR hydrochloride has a high purity and provides a reliable theoretical basis for the industrial production of water-soluble drug sustained-release injection. | (Jin, Q. et al., 2011) |
|  |  | BBR hydrochloride liposomes were prepared by active drug loading method, and the liposomes were separated by cation exchange resin method. The liposomes obtained by this method had small particle size and high encapsulation rate, and the encapsulation rate was different with different dosing sequence. The optimal pH value of external water phase is 6.8. | The mixing sequence of blank liposome, NaHCO_3_ solution and BBR hydrochloride solution has a certain effect on the encapsulation rate. | (Deng, Y. H. et al., 2004) |
| β- cyclodextrin inclusion complex | Oligomer consisting of 7 glucose units bound by 1, 4-glucoside bonds. | The dosage of BBR was 0.030g, β-cyclodextrin was 2.0g, the inclusion time was 90min. | The antibacterial ability of liposome is stronger than BBR alone, which can reduce drug dose and stimulation, prolong drug action time and improve drug efficiency, which has great economic and practical value in pharmaceutical engineering. | (Li, Z. P. et al., 2003) |
|  |  | It was prepared by saturated aqueous solution method and orthogonal test method. The optimum process was as follows: temperature was 80℃, inclusion 2h, and the ratio of host and guest molecules was 4∶1 (g/g). | It can improve the solubility of insoluble drugs, improve bioavailability and cover up odors. | (Qi, L. M., 2010) |
| Dropping pill | drug is heated and mixed with the matrix, insoluble condensing agent is dropped, and the molten drop shrinks into a pill in the condensate and then condenses into a solid state. | PEG1000+PEG4000 (1∶1) was used as the matrix, the drug-matrix was 1∶4, the material temperature was 95℃, the dimethylsilicone oil was used as the coolant, the coolant temperature was 5℃, the drip diameter was 3mm, the drip rate was 50 drops /min, the drop distance was 6cm. | This process provides a reference method for reforming some insoluble drug dosage forms. | (Chen, S. B. and Zhou, L. J., 2008) |
|  |  | The ratio of drug and matrix (PEG6000) is controlled at 1∶4, the drop rate in the drip process is controlled at 40 or 50 drops /min, the temperature of the liquid is 70 or 85 ~ 90℃, the temperature in the middle of the condensing tube is 6 ~ 8℃, the temperature in the bottom of the condensing tube is -2℃, the drop distance is 5 or 7cm, and the diameter of the dropper is 1.2 ~ 1.5mm or 2mm. The height of condensing column is 90 ~ 100cm. | The prepared dropping pills have small dissolution time, good appearance quality and small difference in pill weight, which meet the quality requirements of dropping pills. The method has certain value for the industrial production and clinical application of dropping pills. | (Luo, Y. N. and Qin, S. M., 2011) |
| Microcapsules | The solid, liquid or gaseous substance is coated in a small, closed system by means of physical chemistry, chemistry and other methods. | Polyacrylic acid resin Ⅳ was dissolved in an appropriate amount of acetone, the raw material was added (1:1), and the suspension drops were added to an appropriate amount of liquid paraffin, stirred to heat up, filtered, and the formed microcapsules were obtained by washing and drying. | Improving drug bitterness without altering pharmacokinetic properties, simple preparation process, large drug load, high encapsulation rate, stability, and good industrial application prospect. | (Liu, J. et al., 2004) |
|  |  | BBR-hydroxypropyl methylcellulose phthalate (HPMCP) (1:7), HPMCP-acetone ethanol mixture (1:20), acetone ethanol mixture - liquid paraffin wax (1:5), sorbitan oleate - liquid paraffin (6:100). | It can reduce the frequency of administration, increase the adaptability of animals to drugs, and avoid drug inactivation in the stomach and reduce stomach irritation. | (Wang, J. et al., 2014) |
|  |  | The mass ratio of core to material was 1∶3, the mass fraction of acacia and gelatin were both 2.50%, the stirring speed was 200 r/min, and the temperature was 53℃. | Improve the taste and reduce the stimulation to gastric mucosa.But the drug carrying capacity of microcapsules is lower than fluidized bed coating. | (Yu, Y. et al., 2015) |
| Microspheres | A spherical or sphere-like entity in which a drug is dissolved and dispersed in a matrix skeleton made of polymer materials. | The volume ratio of anhydrous ethanol to liquid paraffin was 1:8, the mass fraction of sorbitan oleate was 2%, the dosage of BBR was 800mg, the dosage of ethyl cellulose and carbomer were 500mg, and the mass ratio of ethyl cellulose and carbomer was 1:1. | slow release | (Jiang, H. Y. et al., 2015) |
|  |  | The dosage of BBR and polylactic acid was 0.02∶0.3. Water and oil phase volume ratio of 10∶100. The concentration of emulsifier PVA is 3%. | Improve the encapsulation rate of BBR, Improve drug efficacy. | (Liu, Y. J. and Zhang, L. Y., 2008) |
| Pellets | Spherical, spheroid particle size is less than 2.5mm, composed of powder and excipients multi-drug release system. | The solution of sodium alginate of BBR hydrochloride, sodium bicarbonate and chitosan is added to the solution of calcium chloride containing acetic acid. | Can float in the stomach; Slow release. | (Su, Q. et al., 2023) |
|  |  | 1% carboxymethylcellulose sodium (CMC-Na) is the wetting agent, the extrusion rate is 40r/min, the rotating speed is 800r/min, and the rotating time is 2.5min. | The process is simple, the production efficiency is high, the repeatability between batches is good, and the batch preparation can be scaled up, and it is suitable for the drug with less water solubility. | (Yu, L. M., 2012) |
| Nanoemulsion | Particle size of 10~100nm ,the emulsion droplets are dispersed in another liquid to form a colloidal dispersion system. | BBR hydrochloride nanoemulsion was prepared by pseudo ternary phase diagram with isopropyl myristate as the oil phase, polyoxyethylene castor oil as the surfactant and glycerin as the cosurfactant. | Compared with traditional tablets, capsules and aqueous solutions, it has higher antibacterial activity against Escherichia coli, Salmonella, Staphylococcus aureus and Streptococcus agalactis, reduces the dose and stimulating effect of BBR hydrochloride, prolongs the action time of the drug and improves the therapeutic effect of the drug. | (Sun, H. W. and OY, W. Q., 2007) |
| Nanoparticles | A novel drug carrier with a particle size of 1 ~ 1000nm. | Gelatin was 10g/L, the volume fraction of coagulant was 81.25%, the titration rate was 2ml/min, the stirring rate was 600r/min, the mass ratio of BBR hydrochloride to gelatin was 2∶4, and the volume fraction of crosslinking agent was 10%. | slow release | (Li, X. D. et al., 2015) |
|  |  | A sodium tripolyphosphate solution with a concentration of 1.5mg·mL-1 was slowly dropped into a chitosan solution with a concentration of 0.5mg·mL-1 BBR, and stirred for 10 minutes to obtain BBR chitosan nanoparticles. | Improve drug bioavailability | (Lin, A. H. et al., 2007) |
| Tablet | Orally disintegrating tablets | BBR hydrochloride was coated with acrylic resin, and the mass ratio of drug to coating material was 1:0.8. The drug microcapsules formed by coating were then pressed into tablets with 6% crospovidone and 15% microcrystalline cellulose tablets. | Conceal the bitterness of BBR, easy to use, fast disintegration speed. | (Hu et al., 2013) |
|  | Gastric floating tablets | Weigh BBR and various auxiliary materials according to the prescription, grind and pass 80 mesh sieve respectively, fully mix with equal amount method, add magnesium stearate, mix well. Press the tablet, the hardness of the tablet is controlled at 5kg. | It has good floating characteristics and drug release performance, and the production process is simple, and may be an alternative dosage form for the treatment of gastrointestinal diseases. | (Ji et al., 2017) |
|  | Colon-location tablets | The multi-layer film coating technology of rolling coating machine was adopted. Hydroxypropyl methylcellulose was used as isolation layer, PH-sensitive acrylic resin mixed coating solution was used as enteric-soluble layer, permeable acrylic resin mixed solution was used as slow-release layer, triethyl citrate was used as plasticizer and talc powder was used as anti-stick agent. Isolation layer weight increased by 1.2%; Enteric layer composition (1:5), coating weight increased by 4%; Slow-release layer composition (1:1), coating weight increase 2%. | It can improve the operability and is suitable for industrial production, and makes a beneficial exploration for the further study of colon targeted drug delivery system of traditional Chinese medicine. | (Liu, X. et al., 2008) |
|  |  | An appropriate amount of microcrystalline cellulose was added to BBR hydrochloride, pectin and guar gum enzyme-controlled skeleton materials were pressed into a skeleton core and then coated with intestinal coating to make a colon-positioned skeleton coated tablet. The ratio of pectin to guar gum was 1:1, and the weight of enteric coating was increased by 3.8%. | It can make the drug release less than 20% in artificial intestinal fluid in 5h, and close to 80% in simulated colon environment in 6h. | (Xiao, Y. et al., 2008) |
| Gels | Ophthalmic | Appropriate amount of BBR hydrochloride and sodium chloride were dissolved in distilled water, Poloxamer was added (25% Poloxamer 407 and 4.19% Poloxamer 188), completely dissolved, and 0.02% benzalkonium bromide was added to make BBR hydrochloride ophthalmic gel. | The problems of short retention time and low bioavailability of conventional ophthalmic liquid preparations were solved. The problems of poor spreading and difficult dose control of common ophthalmic gel were overcome. | (Hao, J. F. et al., 2010) |
|  | Nasal | Weigh BBR hydrochloride, add Tween-80 in the mortar, homogenize, add glycerin and 0.2% Carbomer-980 aqueous solution, add 10% (V/V) triethanolamine solution, adjust pH6.0 ~ 7.0, quantify, stir evenly, vacuum debubbling for 12h. | simple prescription, stable quality and release | (Wang, L. F. et al., 2014) |
| Powders |  | The optimum process of BBR hydrochloride/montmorillonite composite powder is as follows: feed ratio 1:3, temperature 70℃, reaction time 2h. | It has good inhibitory effect on Escherichia coli. | (Sun, M. et al., 2017) |

**Supplementary Table 3.** Targets of direct BBR action

| **Direct target of berberine** | **Disease** | **Technology** | **Significance** | **Reference** |
| --- | --- | --- | --- | --- |
| EIF2AK2, eEF1A1, PRDX3, and VPS4B | Inflammatory | ABPP | It may be an important therapeutic target for inflammation-related diseases | (Wei et al., 2023) |
| NEK7 protein | Inflammatory | ABPP | New inhibitors of NEK7-NLRP3 interaction may be developed. | (Zeng et al., 2021a) |
| FtfL | Colorectal cancer | ABPP | Dissect related mechanisms that prevent the occurrence and development of colorectal cancer from the perspective of intestinal microorganisms. | (Yan et al., 2023) |
| RXRα | Colorectal cancer | Luciferase assay, Lentiviral vector-based shRNA technique, Isothermal titration calorimetry, etc | Develop new strategies for designing a new RXRα-based anticancer agents and medication combinations. | (H et al., 2017) |
| UHRF1 | Multiple myeloma | SPR, LC-MS/MS | It is helpful for the treatment of multiple myeloma by BBR | (Gu et al., 2020) |
| MAP2K7 | Obesity, Neurodegenerative diseases, etc. | ABPP, CETSA | Propose MAP2K7 as a druggable target for the development of selective JNK pathway modulators. | (Zeng et al., 2021b) |
| Actin | Cancer | An affinity-based chemical probe of berberine, Mass spectrometry | It provides a reasonable explanation that BBR inhibits cell migration and cancer cell invasion. | (Yi et al., 2017) |

## Supplementary Figures


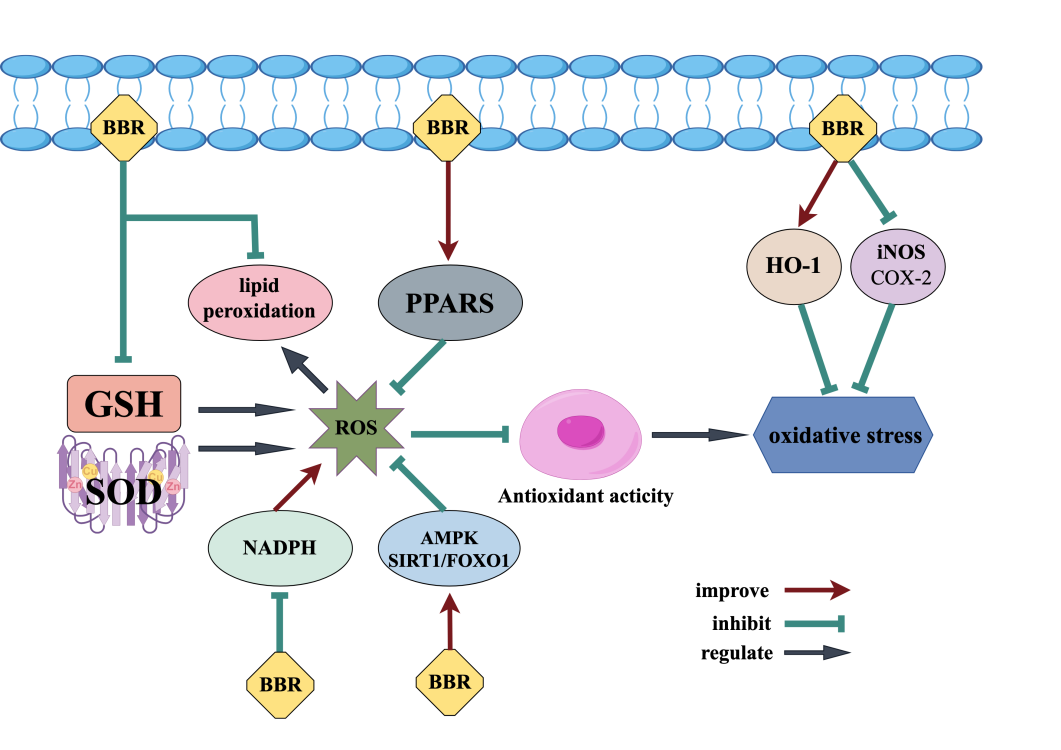


**Supplementary Figure 1.** Mechanism of BBR against oxidative stress

**
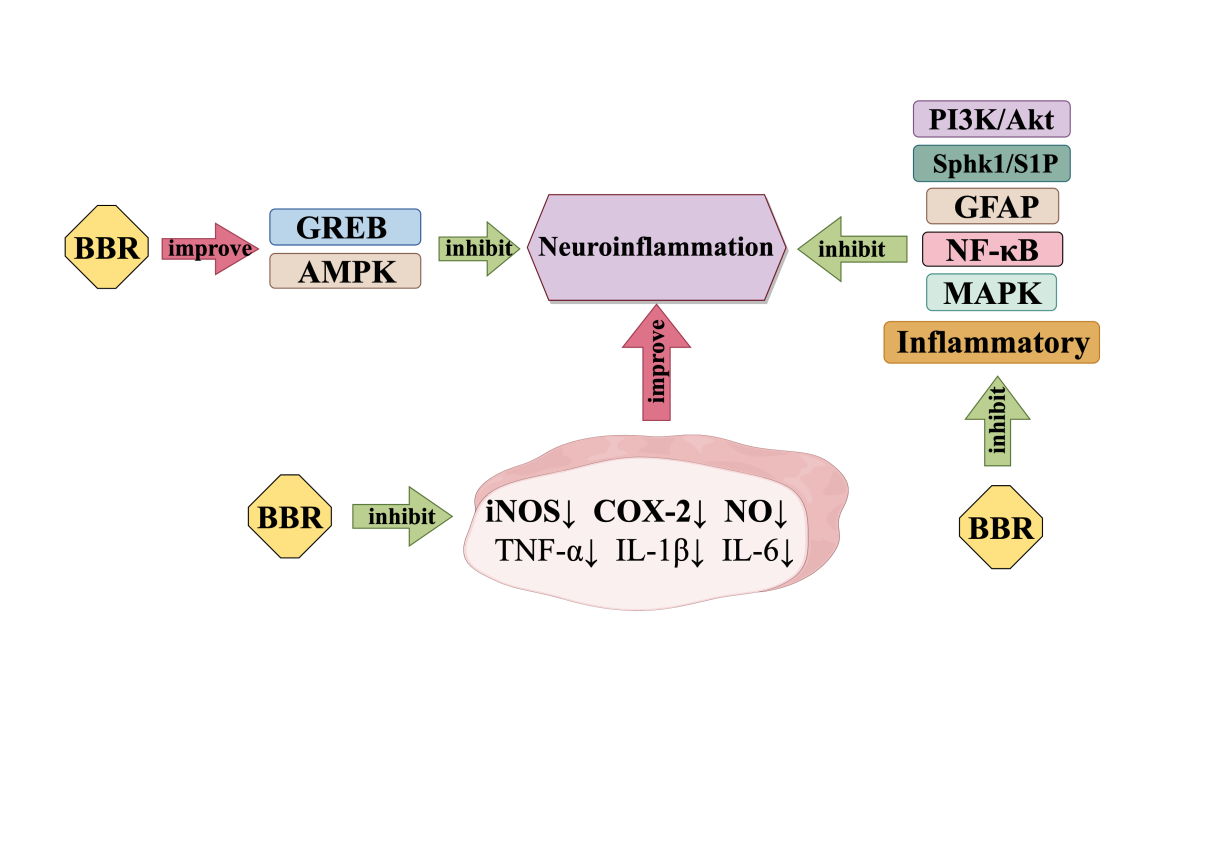
**

**Supplementary Figure 2.** Anti-neuroinflammatory mechanism of BBR

**
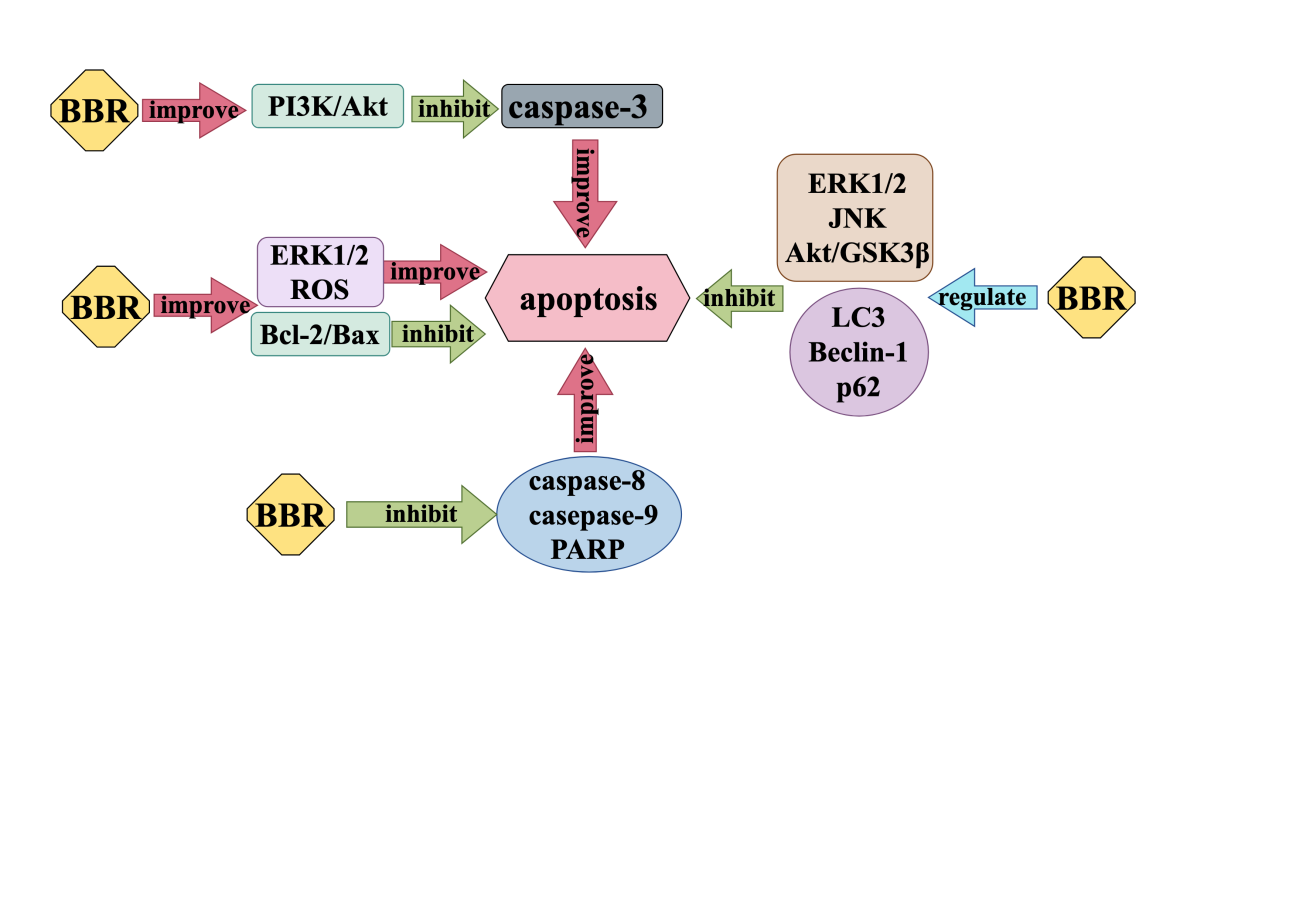
**

**Supplementary Figure 3.** Regulation of apoptosis mechanism of BBR

**
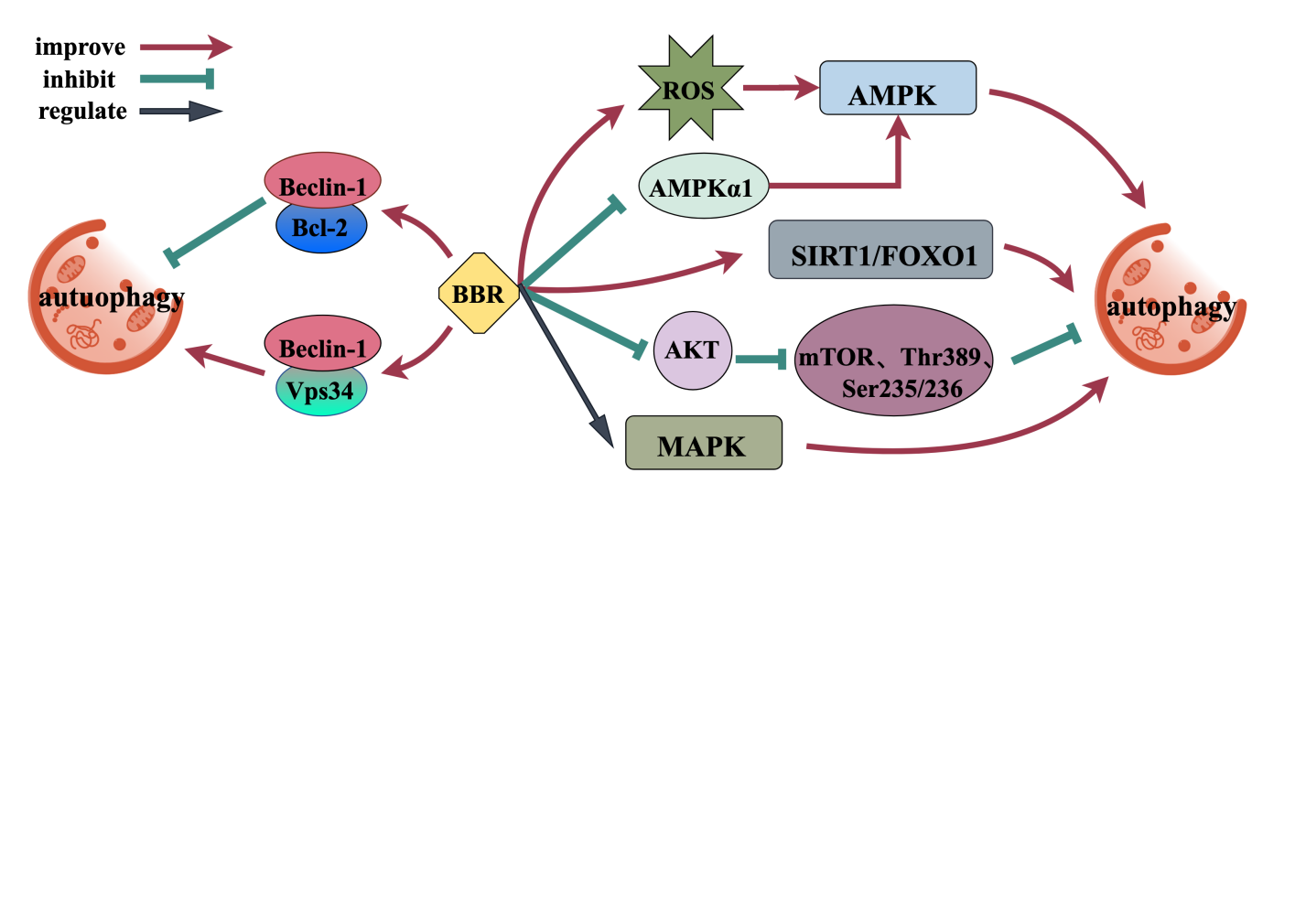
**

**Supplementary Figure 4.** Mechanism of BBR regulating autophagy

**
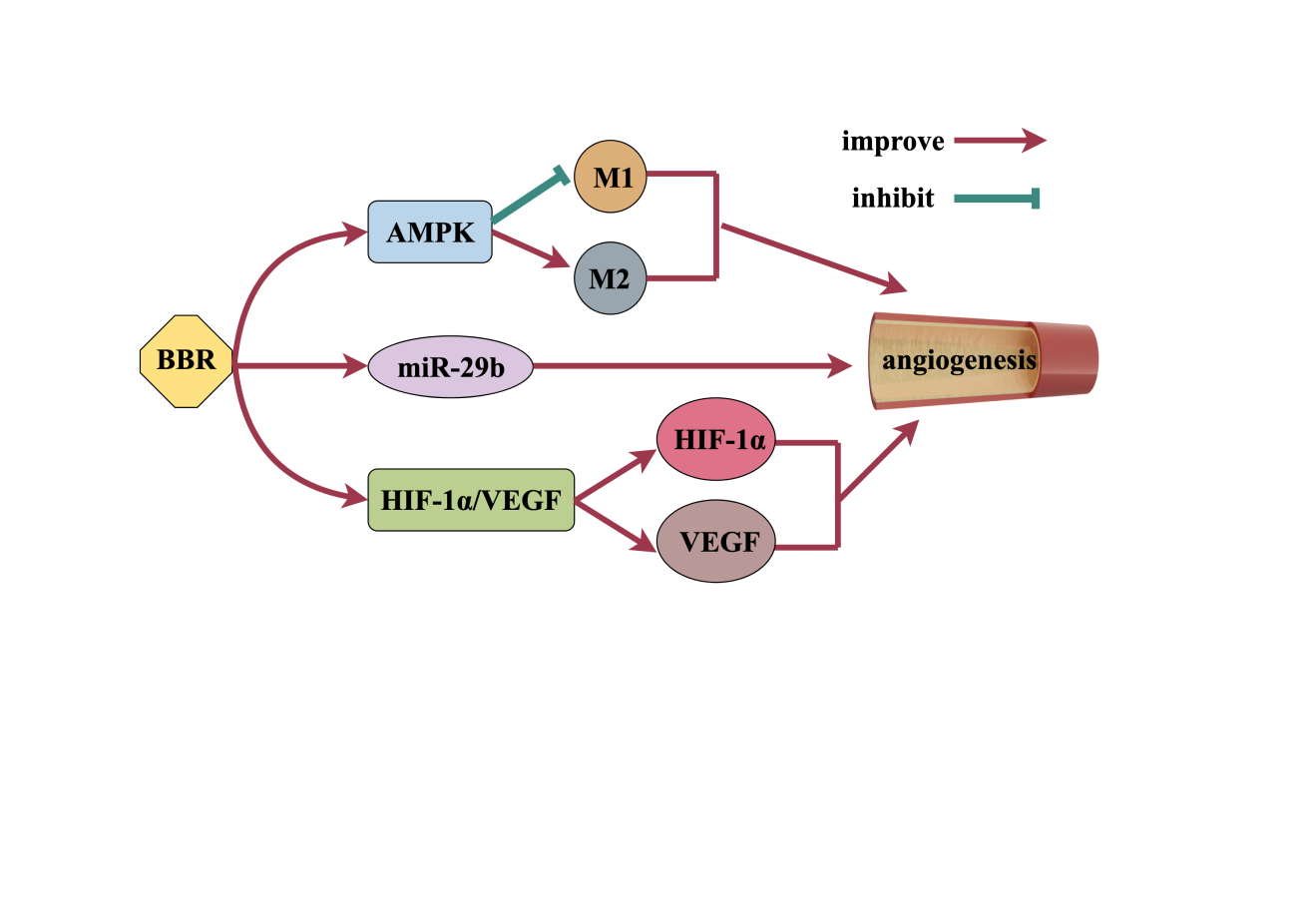
**

**Supplementary Figure 5.** Mechanism of BBR promoting angiogenesis

**
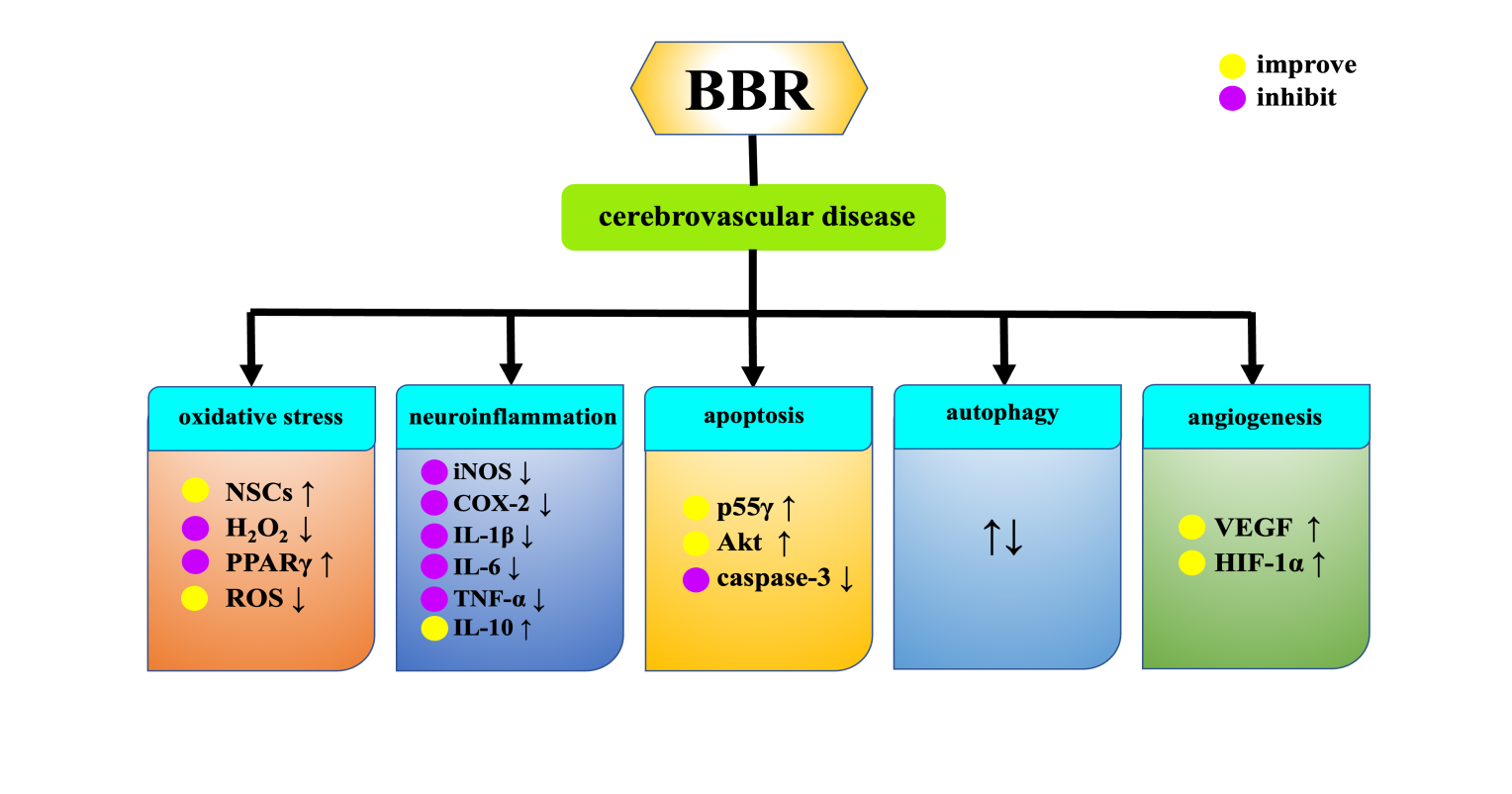
**

**Supplementary Figure 6.** Effects of BBR on cerebrovascular diseases


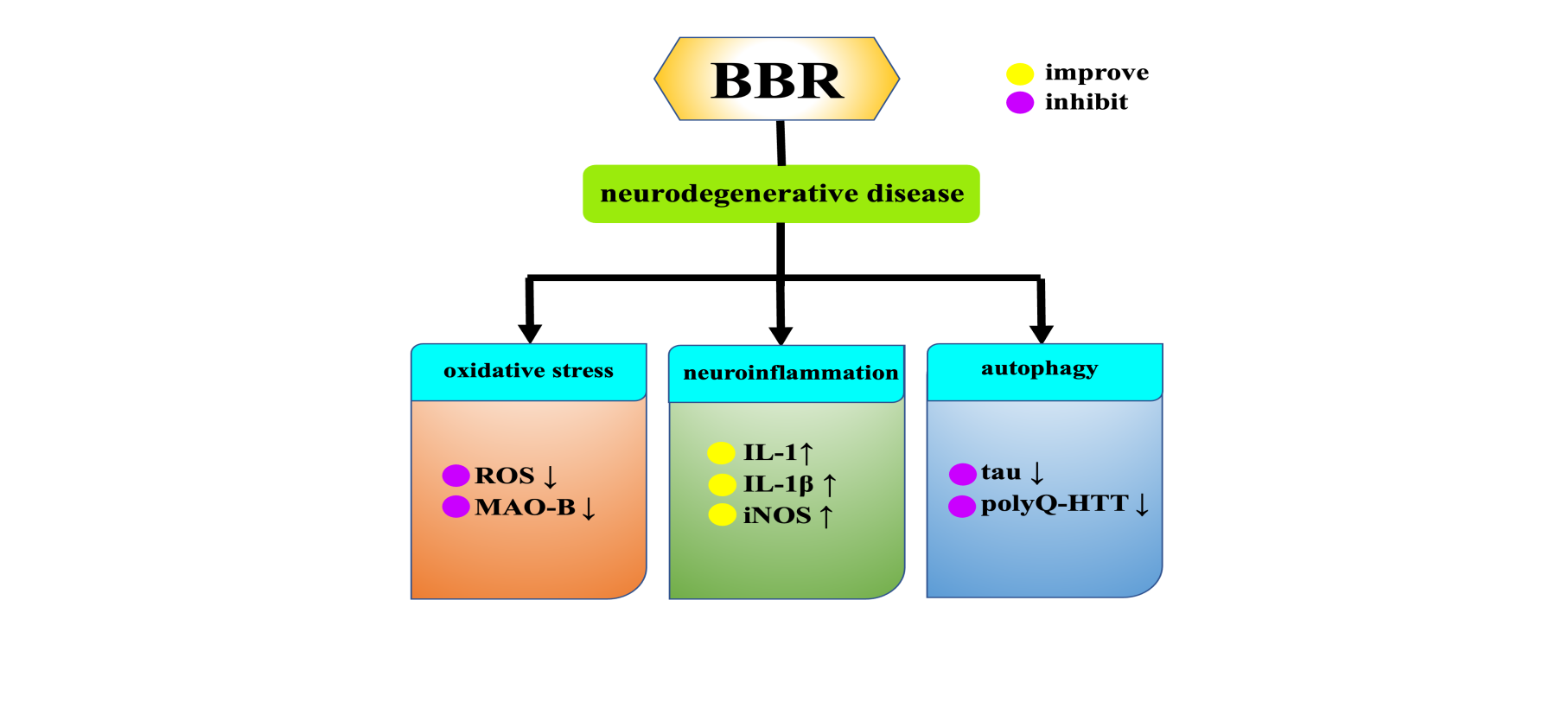


**Supplementary Figure 7.** Effects of BBR on neurodegenerative diseases


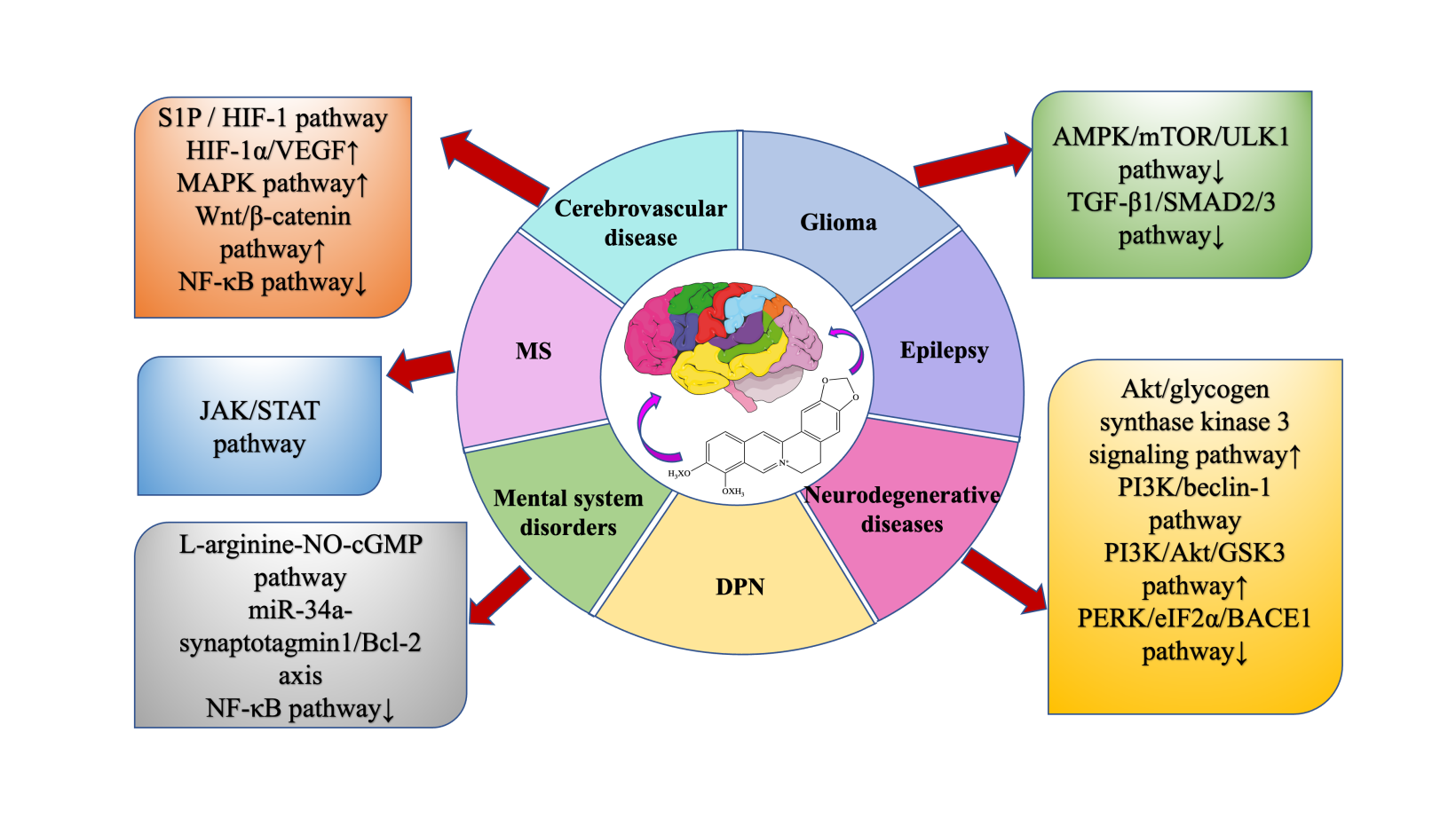


**Supplementary Figure 8.** Neuroprotective pathway of BBR

**Supplementary Figure 9.** Effects of BBR on cerebrovascular diseases
